# Encephalitis in Patients with COVID-19: A Systematic Evidence-Based Analysis

**DOI:** 10.3390/cells11162575

**Published:** 2022-08-18

**Authors:** Md Asiful Islam, Cinzia Cavestro, Sayeda Sadia Alam, Shoumik Kundu, Mohammad Amjad Kamal, Faruque Reza

**Affiliations:** 1 Department of Haematology, School of Medical Sciences, Universiti Sains Malaysia, Kota Bharu 16150, Malaysia; 2 Institute of Metabolism and Systems Research, University of Birmingham, Birmingham B15 2TT, UK; 3 Headache Centre, Department of Neurology, San Lazzaro Hospital, ASL CN2, 12051 Alba, CN, Italy; 4 Department of Biochemistry and Molecular Biology, Jahangirnagar University, Savar, Dhaka 1342, Bangladesh; 5 Institutes for Systems Genetics, Frontiers Science Center for Disease-Related Molecular Network, West China Hospital, Sichuan University, Chengdu 610064, China; 6 King Fahd Medical Research Center, King Abdulaziz University, Jeddah 22230, Saudi Arabia; 7 Department of Pharmacy, Faculty of Allied Health Sciences, Daffodil International University, Dhaka 1207, Bangladesh; 8 Enzymoics, 7 Peterlee Place, Novel Global Community Educational Foundation, Hebersham, NSW 2770, Australia; 9 Department of Neurosciences, School of Medical Sciences, Universiti Sains Malaysia, Kota Bharu 16150, Kelantan, Malaysia

**Keywords:** COVID-19, coronavirus, SARS-CoV-2, encephalitis, meningoencephalitis, encephalopathy, systematic review

## Abstract

Although severe acute respiratory syndrome coronavirus 2 (SARS-CoV-2) predominantly infects the respiratory system, several investigations have shown the involvement of the central nervous system (CNS) along the course of the illness, with encephalitis being one of the symptoms. The objective of this systematic review was to evaluate the characteristics (clinical, neuro-radiological aspects, and laboratory features) and outcomes of encephalitis in COVID-19 patients. PubMed, Scopus, and Google Scholar databases were searched from 1 December 2019 until 21 July 2022 to identify case reports and case series published on COVID-19 associated with encephalitis. The quality of the included studies was assessed by the Joanna Briggs Institute critical appraisal checklists. This systematic review included 79 studies, including 91 COVID-19 patients (52.7% male) experiencing encephalitis, where 85.6% were adults (49.3 ± 20.2 years), and 14.4% were children (11.2 ± 7.6 years). RT-PCR was used to confirm 92.2% of the COVID-19 patients. Encephalitis-related symptoms were present in 78.0% of COVID-19 patients at the time of diagnosis. In these encephalitis patients, seizure (29.5%), confusion (23.2%), headache (20.5%), disorientation (15.2%), and altered mental status (11.6%) were the most frequently reported neurologic manifestations. Looking at the MRI, EEG, and CSF findings, 77.6%, 75.5%, and 64.1% of the patients represented abnormal results. SARS-CoV-2-associated or -mediated encephalitis were the most common type observed (59.3%), followed by autoimmune encephalitis (18.7%). Among the included patients, 66.7% were discharged (37.8% improved and 28.9% fully recovered), whereas 20.0% of the reported COVID-19-positive encephalitis patients died. Based on the quality assessment, 87.4% of the studies were of high quality. Although in COVID-19, encephalitis is not a typical phenomenon, SARS-CoV-2 seems like a neuropathogen affecting the brain even when there are no signs of respiratory illness, causing a high rate of disability and fatality.

## 1. Introduction

The severe acute respiratory syndrome coronavirus 2 (SARS-CoV-2) virus attacks the respiratory system. According to the World Health Organization’s most recent data, over 550 million individuals have been infected, with over 6 million deaths globally [1]. As the coronavirus disease 2019 (COVID-19) pandemic continues, there is growing evidence that this virus also affects the central nervous system (CNS), exhibiting its potential neurotropic and neuroinvasive properties [2,3]. Besides systematic, respiratory, and gastrointestinal symptoms, [4,5,6], neurological manifestations are increasingly recognized in patients with COVID-19, including headache, smell dysfunction, taste disorder, and seizure [7,8,9]. Published data have been suggesting that encephalitis is one of the most fatal neurologic manifestations of COVID-19 involving both adult and pediatric patients [10,11,12]. While the exact mechanism of CNS invasion is still being investigated, possibilities have included both direct viral invasion and indirect damages via inflammatory and autoimmune pathways [13,14].

Encephalitis is an inflammation of the brain parenchyma, exerting serious neurological dysfunction, which is majorly caused by viruses characterized by clinical manifestations such as confusion, reduced or alternative levels of consciousness, fever, headache, seizures, and movement disorder. Diagnosis of encephalitis is usually a combinational approach of laboratory, neuroimaging, and electrophysiologic findings, including blood tests, bronchoalveolar lavage or sputum, urine and stool tests, computed tomography (CT) scan, X-ray, electroencephalogram (EEG), lumbar puncture, and magnetic resonance imaging (MRI) [15,16].

The first case of COVID-19-associated meningoencephalitis was confirmed in a 24-year-old male with severe febrile confusion and generalized tonic-clonic seizure in February 2020 [17]. A recent multicenter retrospective study conducted by the Spanish Society of Neurology reported that encephalitis was present in 2.2% of the COVID-19 patients with neurological symptoms [18]. Previous studies have reported several clinical and laboratory features of SARS-CoV-2-mediated encephalitis. As there has been an escalating number of incidents of encephalitis in COVID-19 patients, with alarming morbidity and mortality rates, this study aimed to systematically evaluate the characteristics (clinical, neuro-radiological aspects, and laboratory features) and outcomes of encephalitis in COVID-19 patients, as well as the possible causative mechanisms of CNS damage.

## 2. Methods

### 2.1. Study Guideline

This systematic review (PROSPERO registration number CRD42022354224) implemented the updated Preferred Reporting Items for Systematic Reviews and Meta-Analyses (PRISMA) 2020 guideline [19].

### 2.2. Search Strategies

We used the advanced and expert options of PubMed, Scopus, and Google Scholar databases, searching for journal articles published between 1 December 2019 and 21 July 2022, combining appropriate keywords associated with COVID-19 and encephalitis (Table S1).

### 2.3. Eligibility Criteria

We considered only case reports or case series published in the English language. In this systematic review, cases with confirmed encephalitis or meningoencephalitis were considered eligible; however, cases reporting only meningitis were excluded.

### 2.4. Study Screening and Selection

Following the removal of duplicate studies using EndNote X8 software (Clarivate Analytics, Philadelphia, PA, USA), titles, abstracts, and full texts were screened independently by two authors (MAI and SSA) to identify the eligible studies. The reference lists of the included studies were also reviewed to identify any potentially eligible studies. Any disagreements on whether a study should be included or excluded were discussed with the third author (CC) and resolved subsequently.

### 2.5. Data Extraction

From each study, all the important data and information was retrieved by two authors (MAI and SSA), including study ID (first author’s last name and year of publication), country of origin of the patient, number, age and gender of the patient, SARS-CoV-2 confirmatory test, past medical history, severity of COVID-19, onset of encephalitis from COVID-19 presentation, neurological and psychiatric symptoms, type of suspected or confirmed encephalitis, patients’ outcome, leukocyte types present, opening pressure when performing lumbar puncture, serum blood glucose value, IgG index, brain computerized tomography (CT) scan, magnetic resonance imaging (MRI) and electroencephalogram (EEG) results, white blood cells (WBCs) level, total protein and glucose concentration, status of SARS-CoV-2, and investigation of other pathogens. All the extracted data were verified by another author (CC).

### 2.6. Quality Assessment

The Joanna Briggs Institute (JBI) critical appraisal checklist for assessing case reports and case series were used to evaluate the methodological quality of the included studies. Two authors (SSA and SK) independently assessed the quality of each of the included studies, and discrepancies were resolved by discussing with the third author (MAI). Studies receiving scores of <50, 50–70, or >70% were classified as low quality (high risk of bias), moderate quality (moderate risk of bias), or high quality (low risk of bias) [9].

## 3. Results

### 3.1. Study Selection

**Figure 1:**
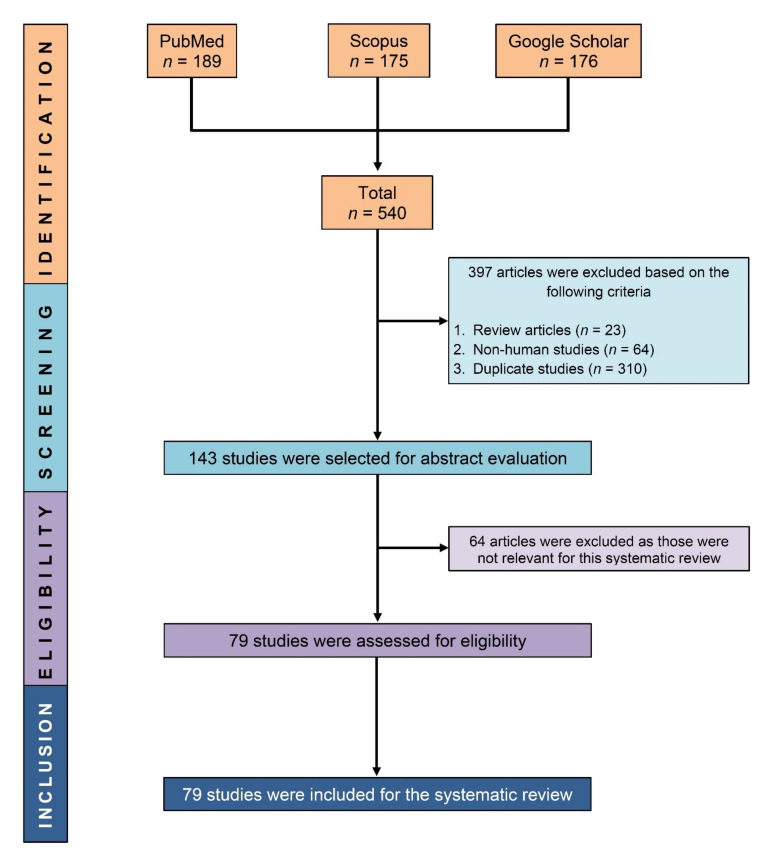
PRISMA flow diagram showing the process of selecting eligible studies.

After excluding review articles (*n* = 23), non-human research (*n* = 64), and duplicate studies (*n* = 310) from our initial search results (*n* = 540), 143 papers were evaluated for eligibility, and 79 studies were eventually included in this systematic review (Figure 1).

### 3.2. General Characteristics of the Included Studies

**Figure 2:**
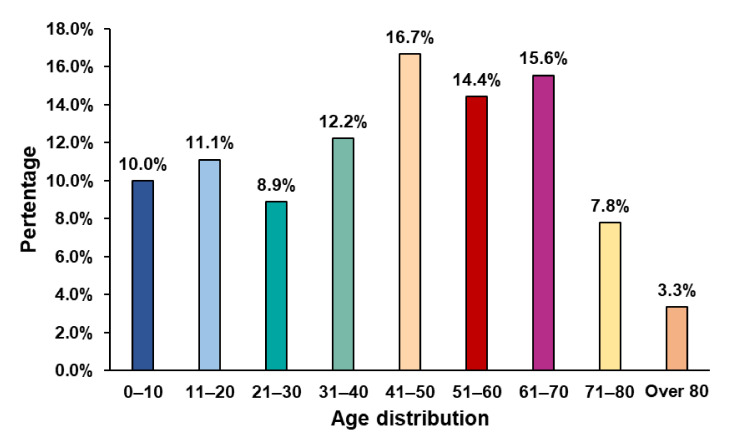
Age distribution of COVID-19 patients with encephalitis.

Major characteristics of the included studies are presented in Table 1. In brief, among the included 91 patients, 52.7% were male, 85.6% were adults (aged 49.3 ± 20.2 years), and 14.4% were pediatric patients (aged 11.2 ± 7.6 years), where a majority of the patients’ ages ranged between 41 and 70 years (Figure 2). The majority of the patients’ SARS-CoV-2 (92.2%) were confirmed by using the RT-PCR method.

A majority of the studies were reported on European (37.2%), followed by Asian (30.8%) and North American (23.1%), COVID-19 patients. Of these patients, 31.9 were healthy, without any past medical history of illness. Among the reported levels of severity of the COVID-19 patients, 11.7%, 38.3%, 11.7%, and 38.3% of the patients were denoted as asymptomatic, mild, moderate, and severe, respectively. Interestingly, the onset of encephalitis-associated symptoms was manifested in 78.0% of the COVID-19 patients as an initial presentation. Seizure (29.5%), confusion (23.2%), headache (20.5%), disorientation (15.2%), and altered mental status (11.6%) were the most frequently reported neurologic manifestations. Among the types of diagnosed encephalitis, a majority were confirmed to be SARS-CoV-2-associated or -mediated encephalitis (59.3%), followed by autoimmune encephalitis (18.7%). Among the included patients, 66.7% were discharged (37.8% improved, 28.9% fully recovered, and 12.2% patients were discharged without reporting their outcomes), whereas 20.0% of the reported COVID-19-positive encephalitis patients died. Looking at the MRI, EEG, and CT scan findings, 77.6%, 75.5%, and 64.1% of the patients presented with abnormal results, respectively. All the diagnostic features of the COVID-19 patients with encephalitis are presented in Table 2 and S2.

### 3.3. Evidence-Based Analyses

#### 3.3.1. Infant to Children with COVID-19

Seizures were frequently present, and prognosis was often poor. Neurologic involvement in a term neonate with prenatal exposure to SARS-CoV-2 was described for the first time in 2022 [62]. The main and first symptoms of this baby’s infection were respiratory distress and ground glass pneumonia. She manifested seizure thereafter, and neurological deficit only at 49 days of disease following extubating and respiratory improvement. The neurological deficit corresponded to documented MRI lesions. Only IG therapy showed some benefit. A fatal case of acute hemorrhagic necrotizing encephalitis (AHNE) affected a 2-month-old boy, who presented with fever and general symptoms, but had cardiac arrest followed by brain death within a few days [66]. Another young patient was a 9-month-old infant [84]. The initial symptoms were fever and profuse vomiting for two days. Then, he manifested convulsive seizure and consciousness alteration. CT scan indicated severe hydrocephalus, and a high protein titer in the CSF suggested aseptic meningoencephalitis (Table 1). Despite antibiotic, antiviral, anticonvulsant, and anti-edematous medication, the child had a cardiac arrest and had to be resuscitated and intubated. Due to migrating to another country, the rest of the history of the infant remained unclear. A 34-month boy also had a poor prognosis [35]. He manifested with fever, seizure, and upward gaze, with progressive worsening, and was admitted to intensive care unit due to recurrent seizures and consciousness compromission. His brain MRI documented scattered foci of altered signal together with hemorrhagic foci. Therapy based on antibiotics, antiviral, and antiepileptic drug (AED) failed; he had a mild improvement after dexamethasone, hydroxychloroquine, and intravenous immunoglobulins (IVIG). He was finally in a vegetative state. Six more children—three girls, one aged 23 months [31] and two others aged 7 years [20,55], and two boys aged 7 and 11 years [11,46]—were described with febrile and manifesting epileptic seizures. Ahsan’s and McAbee’s [11,20] cases were with status epilepticus. Burr’s [31] child also showed neurological deficits with language disorders and involuntary movements. Neurological impairment occurred up to nine days after the onset of symptoms; an MRI of the brain revealed no abnormalities. Anti-NMDAR receptor antibodies were detected both in serum and CSF [31]. In this case, the initial treatment with IV methylprednisolone was unsuccessful; therefore, IV immunoglobulin (IVIG) was given instead. The case described by Ahsan et al. [20] tells a similar story, with status epilepticus and aphasia following sporadic self-limited seizures in the prior week and neurologic worsening one week later. This girl’s MRI documented alteration in the perirolandic and posterior area of the parietal lobe, with cortical edema. Following the investigation of various autoantibodies, anti-myelin oligodendrocyte glycoprotein (MOG) antibodies were detected. Following that, IVIG therapy was given, and the child improved over the following five days before being sent home; mild dysarthria persisted at follow-up. In addition, the boy described by Ferdosian et al. [46] had fever and aphasia, with consciousness alteration, for three days, and a history of recurrent seizures in the last five months. His MRI documented diffuse brain edema and CSF contained SARS-CoV-2 RNA. He improved initially with therapy with antibiotics, antiviral, and AED, but did not improve after therapy with IVIG, remdesivir, and AED.

The child described by McAbee et al. [11], who developed status epilepticus and fever after two days of generalized weakness, achieved a better prognosis. He was treated with anti-epileptic drugs (AED), and recovery was completed in six days. A girl, described by Kahwagi et al. [55], was the only child manifesting respiratory symptoms. She had a cough, headache, and fever for six days, followed on day six by multiple generalized tonic-clonic seizures. Her MRI was unremarkable. She experienced gait and behavioral issues, confusional syndrome, and osteotendinous hyperreflexia on day nine. Her EEG background displayed slowness and superimposed pseudoperiodic complexes. This girl was treated with only AED; her seizures diminished, and behavior disorders disappeared over the next two months. Finally, a five-year-old girl manifested with respiratory symptoms, low-grade fever, and neck edema with lymphadenopathy [87]. In a few days, she became lethargic; her EEG was characterized by slow-wave rhythm, and brain oedema with altered signal of splenium of corpus callosum and subcortical parietal lobes was detected in brain MR. She improved and was discharged after two weeks, after having been treated with antibiotics, antivirals, and steroids.

#### 3.3.2. Adolescents with COVID-19

Seven teenagers suffered from encephalitis attributed to COVID-19 infection. Other than respiratory symptoms, headache, seizures, mood, and conscious level alteration were the main neurologic manifestations. Natarajan et al. [10] reported the case of a 13-year-old girl with a fluctuant history of fever, headache, tonic seizures, and status epilepticus. Although she did not manifest respiratory symptoms, her chest CT scan revealed patchy peripheral ground glass opacities. She was treated with levetiracetam and ceftriaxone, and improved in 48 h. Ground glass opacities in the lungs were also detected in the 16-year-old boy mentioned by Bhavsar et al. [29]. This boy suffered from pharyngitis, headaches, fever, and generalized weakness. On day 11, his neurologic status worsened, with progressive somnolence, confusion, incoherent speech, and walking. After that, he had seizures that required benzodiazepine and AED. Laboratory investigations revealed plasmatic and urinary alterations consistent with the syndrome of inappropriate antidiuretic hormone secretion (SIADH), and his EEG had a slow background. He was treated with antibiotics, and improvement of hyponatremia was accomplished, although he exhibited persistent neurologic disfunction. After one week, he developed an infrapopliteal deep venous thrombosis, and therapy with low-molecular-weight heparin was started. He was discharged to his home on day 15.

An 18-year-old girl was also infected with ground glass pneumonia [21]. She also manifested with seizures and mood disorders. A brain edema and the presence of anti-NMDA receptor antibodies were causative alterations. Steroids and IVIG determined a good prognosis. Two girls with learning disabilities worsened both intellectually/psychically and with neurological deficit. The 19-year-old described by Kasturiachi et al. [57] had progressive encephalopathy, epileptic EEG alteration without seizures, and multiple MR brain lesions. This case was complicated due to coexisting thrombotic thrombocytopenic purpura and other serologic poliautoimmunity; however, her prognosis was good after therapies with plasmapheresis, steroids, and rituximab.

A controversial case of a 16-year-old girl have been described by Gaughan et al. [48]. She had a history of mild learning needs; she complained of sore throat, fever, and psychotic behavior. After five days of IVIG therapy, the condition worsened, presenting mutism, little to no motor activity, incontinence, and being fed via nasogastric tube. Chest X-ray, MRI, and CSF were unremarkable. Two weeks later, following IVIG therapy, anti-GAD antibodies and extractable nuclear antigen appeared in the serum, transitorily. Repeated EEG showed slow activity. The girl improved 4 weeks after initial presentation and was discharged on day 48. The follow-up visit after six months revealed that she is still experiencing memory difficulties and fatigue.

Another 18-year-old girl, whose case has been described by Ayatollahi et al. [23], had fever, fatigue, malaise, and loss of appetite for a week, which progressed to drowsiness and confusion, and finally urinary retention and repeated generalized tonic-clonic seizure. Both CT brain scans and MRI were normal. In the following days, she had new seizures and fluctuating mood alterations, which were resistant to several therapies. Later on, a new MRI showed altered signals in the claustrum, external capsules, and some areas of the adjacent anterior insular cortex. Thereafter, therapy with intravenous methylprednisolone was started, and after three days, she was discharged in good condition. At follow-up, after one month, MRI showed nearly complete resolution of the previously described signal alterations. Seizures did not recur, although the memory deficit persisted. The last young girl mainly had retinal involvement, with subsequent encephalitis of the temporal lobes. Apart from fever, body pain, headache, and nausea with vomiting, the only neurologic symptom was drowsiness, and the prognosis was good [77].

#### 3.3.3. Adults with COVID-19

##### Initial Presentation with Neurological and Respiratory Symptoms

Most cases manifested respiratory symptoms as an early manifestation of SARS-CoV-2 infection. Six cases also manifested neurological symptoms from the beginning of their clinical history [26,27,42,49,69,93]. Several cases developed neurological impairment following respiratory symptoms. The latency between the onset of the infection with respiratory manifestations and the onset of neurological complications varied between 3 days and 41 days [12,24,27,28,30,32,34,36,37,43,45,51,56,58,64,65,70,76,80,81,82,83,85,86,89,90,91,93,94]. Overall, 13 patients were intubated and mechanically ventilated due to respiratory distress; in these cases, the detection of neurological impairment happened when sedation was interrupted, with latency ranging between 4 and 38 days depending on the case [27,32,34,76,80,82,85,93]. In patients whose respiratory distress was less evident, neurological manifestation appeared after a few days in most cases [27,28,30,37,45,56,58,65,89,91,93,94]. In some cases, the latency ranged from over two weeks to 60 days [12,24,27,64,90]. Three patients began their illness with symptoms other than respiratory and developed neurological impairments between 5 and 17 days after the onset of the initial symptoms [22,47,50]. Their clinical presentations consisted of fatigue and malaise for two weeks in one case [47]; progressive diffuse arthralgia and sore throat, followed by fever [50]; and gastrointestinal manifestations in the third case [22]. An onset with neurological presentation was described in a number of reports [17,25,30,33,38,39,41,44,52,53,54,56,59,60,61,68,71,72,73,74,75,78,79,92]. Respiratory complications came later and were symptomatic in three cases [61,75,78]. Even though several patients did not have subjective respiratory difficulties or changes in arterial oxygen saturation, most of them had lung alterations on thorax CT and/or chest X-ray, which were typical of COVID-19 interstitial pneumonia, primarily as ground glass opacities and areas of parenchymal consolidation.

##### Neurological and Psychiatric Manifestations

Sleep disturbances, progressive altered mental status, psychiatric behavioural symptoms, and hallucination have been described by some authors [22,26,37,47,51,52,56,58,63,72,73,75,78,81,86,89,94]; most of these cases are associated with EEG representations [22,26,37,63,74,75], and some with brain neuroimaging alteration [51,52,56,78,81,94]. Neurological deficit referred to language deficit, cognitive deficits, akinetic syndrome with mutism, signs of cortical impairment, seizures, stroke, cerebellar signs, chorea, paralysis, coma, and signs of brain death [17,25,33,36,37,38,39,47,49,51,52,53,58,59,60,64,65,71,72,74,76,78,79,89,92].

Few cases presented with severe epileptic manifestations. Epileptic manifestations occurred from the first phase of infection, evolving into status epilepticus in some patients. Two of these patients died. The first developed intracranial hypertension with diffuse cerebral edema, and cerebellar herniation occurred [79]. The second case manifested a tonic-clonic seizure, followed by cardiac arrest, and was successfully resuscitated, intubated, and transferred to the intensive care unit. In this case, CT and MRI also showed brain edema and cerebellar herniation [45]. A third patient developed status epilepticus that required the patient to be intubated and mechanically ventilated. Chest CT showed interstitial pneumonia. He had an opening high pressure when lumbar puncture was performed, and SARS-CoV-2 RNA was detected in his CSF, while MRI revealed ventriculitis and encephalitis, mainly in the right mesial lobe and hippocampus [17]. The last case reported early onset of drug-refractory epilepsy; MRI and spectroscopy were suggestive for high-grade glioma; after lobectomy, histopathologic diagnosis was of encephalitis [41]. A late-onset status epilepticus with intracranial hypertension ion caused the death of another patient [12]. In this case, the disease had a two-phase evolution. He had a first respiratory phase, with recovery in 2–3 weeks. After 41 days, the fever reappeared, together with severe headache and vomiting. A brain CT scan and MRI showed unilateral hemispheric vasogenic edema with shift of the medial cerebral structures.

##### Particular Case of COVID-Linked Encephalitis

Some cases were marked by identification of specific neural antibodies. Three patients suffered from NMDA encephalitis [68,73,88]. Clinical manifestations were psychotic symptoms and seizures only. All of them improved with immuno-therapies, including IV methylprednisolone, IVIG, therapeutic plasma exchange. Only in the case described by Valadez-Calderon et al. [88], in which anti-NMDAR and anti-GAD65 antibodies were co-expressed, were EEG (slow rhythm) and MRI (MR signal alteration anterior cingulated cortex and temporal lobes bilaterally) both altered.

One patient developed an MOG antibody-associated encephalitis. He had minor clinical manifestations, but mulptiple MRI signal alterations: T2 and FLAIR, mainly with cortical distribution. This patient also improved after IV steroids [40]. Bickerstaff brainstem encephalitis involved three patients: two women and a man. Their main manifestation was truncal and cerebellar disfunction. In the case with anti-GD1 IgG antibodies [25], MRI images were altered (alterations in the caudal vermis and right flocculus of the cerebellum, and contrast enhancement in the floor of the IV ventricle). Improvement and resolution of symptoms were obtained with IV methylprednisolone. In the case with anti-gangliosides (GQ1b, GT1a, and GM1/GT1a), both EEG and MRI were normal. The patient improved after therapy with IV steroids, IVIG, but mainly after plasma exchange. The third case had serum onconeural antibodies against amphiphysin, and MR of brainstem encephalitis; this man also improved after steroidal therapy [59].

Another case of autoimmune encephalitis was described by Grimaldi et al. [50]. This man presented with progressive diffuse arthralgia and sore throat and interstitial pneumonia, and after 17 days, progressive action tremor, cerebellar syndrome, and diffuse myoclonus. EEG showed diffuse slowing, and MRI was normal, but PET with F-FDG showed putaminal, cerebellum, and diffuse cortical hypometabolism, confirmed by whole-brain voxel-based SPM quantification. Antibodies against the nuclei of Purkinje cell, striatal neurons, and hippocampal neurons were found in both serum and CSF. The patient was then treated with IVIG without clinical improvement. He improved after therapy with IV methylprednisolone and clonazepam.

Other particular cases of encephalitis were described. Three case reports described limbic encephalitis [43,76,92]. All of these patients had severe pneumonia, seizures or consciousness alteration, and anti-typical MRI alterations. All of them improved after having been treated with steroids, only in one case with IVIG added. A 67-year-old woman had an acute disseminated encephalitis (ADEM), with drowsiness; she had bilateral pneumonia and respiratory distress, was treated with IV steroids and IVIG, and unfortunately died after 4 weeks [44]. Two cases of mild encephalitis with reversible splenial lesion syndrome (RESLES) were described [42,54]. Both of them had interstitial pneumonia. The first patient [42] was afebrile, and presented a short loss of consciousness, cough, and headache. CSF and brain CT scan were normal, so he was discharged from the hospital. Nine days later, he manifested persistent headache, vertigo and intermittent disturbance of consciousness, myalgia, tiredness and persistent bibasilar rales, psychomotor slowing, and vestibular syndrome. MRI documented a signal alteration on the splenium of the corpus callosum. He was treated with analgesics and antibiotics. The patient improved gradually over the next few days. At one-month follow-up, lung complications were reduced, and MRI normalized. The second patient [54] showed marked dysmetria and mild ataxic gate. MRI documented abnormal alteration of the splenium of the corpus callosum, with a suspicion of RESLES. After a few hours, they manifested fever and hypoxemia. Treated with antiviral, antibiotics, and steroids, they neurologically improved but died at day 12 due to respiratory failure. Seven cases had AHNE [49,69,70,90]. This is a severe condition with poor prognosis. Neurological manifestations appeared after several days but were abrupt. Only four cases had pulmonary compromission [69,70,90]. All seven adults with AHNE died within 3 weeks.

##### Managing Severe Cases

Severe respiratory impairment was the main feature of some cases and required intubation. Benameur et al. [27] described three cases that manifested neurological complications after sedation was discontinued. In one case, cerebral edema with intracranial hypertensive signs was detected with MRI and CSF examination, consistent with encephalomyelitis and superimposed hypoxic ischemia. This woman died when life support was withdrawn. In the second case, profound encephalopathy with multifocal myoclonus was detected, and the patient was neurologically comatose. At lumbar puncture, there was a high opening pressure, suggesting intracranial hypertension. MRI disclosed a signal alteration at the splenium of the corpus callosum. The third case also had a profound encephalopathy without brainstem reflexes. He was treated with hydroxychloroquine, which caused myoclonus that disappeared at the cessation of this therapy. MRI showed signal alteration involving the temporal lobe. In this last case, the IgG anti-S1 receptor-bindle domain was present in serum, and a mild IgM level for SARS-CoV-2 S1 was found in CSF. In these cases, inflammatory molecules have been dosed in CSF, and in one case (the third), IL-8, IL-10, IP-10, and TNFα were increased. In the case series by Cao et al. [32], all five patients were intubated due to acute respiratory distress syndrome. When sedation was withdrawn, one patient was comatose, and the other four were in an unresponsive wakefulness syndrome (vegetative state). Four of them had high IL-6 level in serum, and one patient also had a slight increase of IL-6 in CSF. MRI showed several lesions in supratentorial deep white matter, in the pons, with several multiple small haemorrhagic lesions. Three out of five patients rapidly improved after therapy with intravenous steroids and therapeutic plasma exchange; one patient died. In addition, in the case by Svedung Wettervik et al. [85], the patient was comatose at the wake-up test after intubation. She had brain edema, and microhemorrhages with basal ganglia involvement, in line with acute hemorrhagic leukoencephalitis. An external ventricular drain was positioned, and CSF had fluctuating pressures, with a large amount of white and red cells. IL-6 was high in both plasma and CSF. This patient underwent therapeutic plasma exchange, after which she improved both clinically and in neuroimaging. Chalil et al.’s case developed respiratory distress two weeks from onset [34]. She was intubated, and severe lung complications were observed in chest CT. Therapy was hydroxychloroquine and tocilizumab. Due to the detection of very high D-dimer and ferritin levels, increased CRP, and severe hypoxemia, full anticoagulation started. Extensive bilateral parieto-occipital intraparenchymal hemorrage were observed with intraventricular extension and acute hydrocephalus. On day 15, brainstem reflexes were absent. Heparin was stopped, and external ventricular drain positioned. The authors interpreted this case as a post-infectious acute necrotizing hemorrhagic encephalopathy, due to thalamic involvement, suggested as typical for acute necrotizing hemorragic encephalopathy [34]. The patient was finally extubated, but severe neurologic deficit persisted.

The most prescribed drugs were antibiotics and antiviral agents. Quite often, hydroxychloroquine and high-dose methylprednisolone have been used. Until 2021, therapeutical plasmapheresis and immunosuppressive drugs were rarely prescribed. After the first period of the COVID-19 pandemic, immunomodulating agents, in particular IVIG and tocilizumab, plasmapheresis, and convalescent’s serum, have been used more often, with good response [21,51,59,60,70,81,83,88], except for the most severe cases, like in ANHE.

### 3.4. Quality Assessment

Among the included studies in this systematic review, based on the JBI critical appraisal tools assessing case reports and case series, 87.3% of the studies were of high quality, followed by 11.4% moderate- and 1.3% low-quality studies (Tables S3 and S4).

## 4. Discussion

In this systematic review, we comprehensively investigated the clinical and laboratory features, as well as the outcomes of COVID-19 patients with encephalitis. COVID-19 encephalitis can present before, together, or after respiratory manifestation of the illness. The most frequent neurological manifestations of encephalitis are seizure, confusion, headache, disorientation, status epilepticus, and altered mental status, and the degree of the condition is dependent mostly upon the severity of COVID-19 infection, entity of respiratory damage, and entity of neuronal damage and its site [91,95]. Severity of respiratory impairment and neuronal damage are the factors that seem to determine the worst prognosis. The knowledge of possible para- or post-infectious SARS-CoV-2 encephalitis, and of possible inflammatory or immune mechanisms of neuronal damage, can drive research for better therapies to improve outcomes [27,96].

In most cases, preforming a lumbar puncture can suggest the presence of encephalitic involvement. Brain CT scan in an emergency can be useful only in those cases that manifest with very acute neurological compromission, and can reveal brain edema, endocranial hypertension, massive necrosis, and or hemorrhagic lesions. A rapid intervention, acting to reduce intracranial hypertension, can help give the patient a chance to survive and receive the proper therapeutical efforts [97,98]. In those cases with psychiatric or epileptic presentation, CT scan can be unremarkable. MRI is also useful to detect small lesions, which can sometimes be transitory. In some cases, the only way to reveal neuronal disfunction is to use a neurological functional test, such as PET [99,100]. EEG is mostly aspecific, with slow background activity. Sometime EEG can also reveal epileptic activity of the non-convulsive type [101].

In the case of COVID-19, confirmed or suspected, it is important to consider possible neurological involvement. The clinician should consider any behavioral changes or alterations of consciousness. These can be due to hypoxic damage, hematochemical alteration, or circulatory problems, or they can be COVID-19-related. However, it can be a manifestation of neuronal involvement. The precocity of a specific intervention is essential to preserve neurons, and in case of any suspicion, proper tests and exams should be performed. Sometimes, neurological damage progresses slowly or tardively, and neuro-clinical symptoms can appear several day or weeks after the onset of the first symptoms. In these cases, an autoimmune mechanism of damage can be supposed, and this should drive the therapeutical approach [102,103].

Although the exact pathogenesis of the SARS-CoV-2 virus’s access to the CNS and triggering of various neurological manifestations is still under investigation, researchers have suggested a few mechanisms. SARS-CoV-2 can exert neurological manifestations by two main mechanisms. The first mechanism is the direct CNS infection, where SARS-CoV-2 gain access to the CNS from the peripheral nervous system, as RNA of the SARS-CoV-2 virus has been detected in the CSF of the COVID-19 patients. As in mature human olfactory nerves, ACE2 has not been reported, and ACE2 is most likely present at low levels in neurons [104,105]. SARS-CoV-2 possibly uses other facilitator(s) to access the CNS. One of the plausible routes for SARS-CoV-2 entry in the CNS could be that upon nasal infection, SARS-CoV-2 enters the CNS through the olfactory bulb, facilitated by neuropilin-1 (retrograde axonal transport), the only part of the CNS not protected by dura, causing inflammation and demyelination [106,107,108]. The second mechanism involves the disruption of the blood–brain barrier via SARS-CoV-2-ACE2 receptor-mediated vascular damage following viraemia. In this inflammation-mediated autoimmune-associated mechanism, both innate and adaptive immune systems play a role via upregulated inflammatory mediators exerting cytokine storm syndrome, which may result in acute hemorrhagic necrotizing encephalitis via perivascular demyelination [109,110,111].

Neurological symptoms seem to be the initial sign of illness in certain cases of COVID-19. A few cases, however, had already developed antibodies against SARS-CoV-2, suggesting a previous asymptomatic or pauci-symptomatic COVID-19 disease. That is why it is important, in case of neurological manifestation, to perform both the nasopharyngeal swab for SARS-CoV-2 and the serum test for antibodies against the virus. It is speculated that SARS-CoV-2-mediated encephalitis may present inflammatory injury, edema, and alterations in consciousness in patients with COVID-19 [91,96]. Although viral encephalitis confirmation requires virus isolation in the CSF, due to transient dissemination of SARS-CoV-2 virus and low CSF titre, it becomes extremely difficult to confirm viral encephalitis in COVID-19 [91]. For the management of encephalitis in COVID-19, we observed that IVIG therapy, plasma exchange, and corticosteroids may be useful in the treatment of COVID-19-related encephalitis.

There are some notable strengths of our study. First, this is the first systematic analysis based on the published case reports and series that was represented in a comprehensive way. Second, the search strategy was strong, as we used multiple databases with robust search strategies. Third, from our quality assessment, a majority of the studies were of high quality; therefore, the outcome of this systematic review is reliable, and it is based on high-methodological-quality studies. Nevertheless, there are some notable limitations. First, due to the study pattern, we could not analyze the data through a meta-analysis approach. Second, due to exploring the clinical, laboratory, and neuro-imaging data in a comprehensive way, we only included case reports and case series, and we did not consider any observational studies. Third, we could not retrieve our data of interest from all of the studies, which leads to incomplete data for some of the cases. However, since there is presently very little published information on encephalitis in COVID-19 patients, our analysis provided a strong early insight into the clinical, laboratory, and neuro-imaging characteristics of encephalitis in COVID-19 patients.

In the future, larger sample sizes would aid in determining if the neurological aspects, particularly the relationship with encephalitis, are purely coincidental, or whether there are phenotypes and associations particular to SARS-CoV-2.

## 5. Conclusions

COVID-19 patients with acute characteristic neurological signs such as seizure, confusion, headache, disorientation, status epilepticus, and altered mental status should be evaluated for viral encephalitis immediately, given the present condition of the COVID-19 pandemic. Patients with COVID-19 who are suspected of having encephalitis should have further testing, such as a brain MRI scan, long-term EEG monitoring, and lumbar puncture. The lack of a characteristic CSF profile of viral meningitis/encephalitis, as well as the negative PCR for SARS-CoV-2 virus in CSF, makes the diagnosis of encephalitis caused by the SARS-CoV-2 virus less evident, pointing to a possible autoimmune neuropathogenesis. In the post-acute phase of SARS-CoV-2 infection, it is critical to evaluate the neurological consequences. In this phase, encephalitis should be diagnosed only if there are clinical signs of brain inflammation, such as pleocytosis in the CSF, imaging alterations, focal seizures, or histological alterations. Even if the virus is found in the CSF, encephalitis should not be diagnosed until brain inflammation is present.

## Figures and Tables

**Table 1:** Major characteristics of the included studies.

No.	Study ID, Country [references]	No. of Patient	Age (Years), Gender	Confirmation of SARS-CoV-2	Past Medical History	Severity of COVID-19	Onset of Encephalitis from COVID-19 Presentation	Neurological and Psychiatric Symptoms	Type of Suspected or Confirmed Encephalitis	Outcome
1	Ahsan 2021, USA [20]	1	7, F	Serology test positive (IgG in serum)	Healthy	Asymptomatic	Post-SARS-CoV-2 infection	1st admission: status epilepticus, aphasia, encephalopathy; 2nd admission: headache, encephalopathy, slurred speech, altered mental status	Autoimmune encephalitis	Discharged and follow-up confirmed recovery with mild dysarthria
2	Allahyari 2021, Iran [21]	1	18, F	RT-PCR positive	NR	NR	Initial presentation	Altered mental status, tonic-clonic seizures, confused state, minor meningismus, neck stiffness	Autoimmune encephalitis (SARS-CoV-2-mediated anti-NMDAR encephalitis)	Discharged with full recovery
3	Andrea 2020, Italy [22]	1	79, F	RT-PCR positive	Rheumatoid arthritis	Mild	Initial presentation	Confusion, somnolence, psychomotor retardation, and cephalea	SARS-CoV-2-mediated encephalitis	Complete recovery in 15 days
4	Ayatollahi 2020, Canada [23]	1	18, F	RT-PCR positive	NR	Mild	Initial presentation	Drowsiness and confusion, generalized tonic-clonic seizure, impaired orientation to time and place and recent memory	Autoimmune encephalitis	Near complete resolution of the claustrum hyperintensities following 1 month, no seizures following 7 weeks
5	Ayuningtyas 2022, Indonesia [24]	1	34, F	RT-PCR positive	Obese	Severe	Initial presentation	Reduced consciousness, altered mental status aggressive behavior, seizure, headache	SARS-CoV-2-mediated encephalitis	Discharged with full recovery
6	Ayuso 2020, Spain [25]	1	72, F	RT-PCR positive	Hypertension, hyperlipidemia, depression, and smoking	NR	Post-SARS-CoV-2 infection (after 8 days of discharge)	1st admission: delirium; 2nd admission: dizziness, oscillopsia, inattention, disorientation, unsteadiness, myoclonus, and ataxia	Autoimmune encephalitis (SARS-CoV-2-mediated Bickerstaff encephalitis)	Discharged; after 2 months, very mild unsteadiness was observed
7	Babar 2020, USA [26]	1	20, F	RT-PCR positive	Obesity and anxiety	Mild	Initial presentation	Acute altered mental status, confusion, ageusia, insomnia, hypervigilance, obsessive thinking, and urinary incontinence	SARS-CoV-2-mediated encephalitis	Near complete resolution of the neurological symptoms after 12 days of discharge
8	Benameur 2020, USA [27]	3	31, F	RT-PCR positive	Sickle cell disease	Severe	Initial presentation	Various neurologic manifestations including myoclonus, affected brainstem reflexes, and encephalopathy	SARS-CoV-2-mediated encephalitis	Died
			34, M		Hypertension		Initial presentation			NR
			64, M		Hypertension		Initial presentation			Discharged without major neurologic sequelae
9	Bernard-Valnet 2020, Switzerland [28]	2	64, F	RT-PCR positive	NR	Mild	Initial presentation	Tonic-clonic seizure, disorientation, strong attention deficit, verbal and motor perseverations and bilateral grasping, hyper-religiosity with mystic delusions, visual hallucinations, and non-convulsive status epilepticus	SARS-CoV-2-mediated meningoencephalitis	Resolution of her symptoms after 96 h of admission
			67, F	RT-PCR positive	NR	Mild	Initial presentation	Intense headache, drowsiness, confusion, motor perseverations, bilateral grasping, and aggressiveness	SARS-CoV-2-mediated meningoencephalitis	Discharged after 72 h without major neurologic symptoms
10	Bhavsar 2020, USA [29]	1	16, M	RT-PCR positive	Healthy	Mild	Initial presentation	Initially intermittent headache; day 11: confusion, incoherent speech, seizure, and altered mental status, with inconsistent awareness of time and place	SARS-CoV-2-mediated encephalitis	Discharged after day 15 with improved mental status
11	Bodro 2020, Spain [30]	2	25, M	RT-PCR positive	Healthy	NR	Initial presentation	Headache, left-side paresthesia and ipsilateral paresis, progressing to confusion and agitation	SARS-CoV-2-mediated encephalitis	Fully recovered within 2 days except for amnesia
			49, M	RT-PCR positive	Healthy	Mild	Initial presentation	Anomic aphasia, disorientation, confusion, and agitation		Fully recovered within 3 days except for amnesia
12	Burr 2021, USA [31]	1	23 months, F	RT-PCR positive	Healthy	NR	Initial presentation	Initial: fussiness, poor sleep; day 9: seizure, hyperkinetic movements, and mood lability	Autoimmune encephalitis (SARS-CoV-2-mediated anti-NMDAR encephalitis)	Discharged and fully recovered after 2 weeks
13	Cao 2020, France [32]	5	49, M	RT-PCR positive	Rheumatoid purpura	Severe	Initial presentation	Headache and anosmia	SARS-CoV-2-mediated encephalitis	Improvement after 6 days of immunotherapy
			56, M	RT-PCR positive	Hypertension	Severe	Initial presentation			Improvement after 2 days of immunotherapy
			61, M	RT-PCR positive	Pulmonary sarcoidosis and thrombocytopenia	Severe	Initial presentation			Improvement after 7 days of immunotherapy
			37, M	RT-PCR positive	Obesity	Severe	Initial presentation			Died
			77, F	RT-PCR positive	Obesity, hypertension and asthma	Severe	Initial presentation			Vegetative state
14	Casez 2021, France [33]	1	96, F	RT-PCR positive	NR	NR	Initial presentation	At onset: anosmia, dysgeusia; day 3: generalized epileptic seizures, and left hemiparesis	SARS-CoV-2-mediated encephalitis	NR
15	Chalil 2020, Canada [34]	1	48, F	RT-PCR positive	Healthy	Severe	Initial presentation	Altered mental status	SARS-CoV-2-mediated acute hemorrhagic encephalitis	During the report, she was undergoing rehabilitation
16	Cheraghali 2021, Iran [35]	1	34 months, child	RT-PCR positive	Healthy	NR	Initial presentation	Tonic-clonic seizures, and loss of consciousness	SARS-CoV-2-mediated encephalitis	Discharged with decerebrate posture
17	Dahshan 2022, Egypt [36]	1	67, M	RT-PCR positive	Hypertension	NR	Post-SARS-CoV-2 infection (8 days after SARS-CoV-2 infection)	Acute confusion state, behavioral changes, agitation, and one attack of loss of consciousness	Autoimmune encephalitis	Discharged with full recovery
18	Dono 2021, Italy [37]	1	81, M	RT-PCR positive	Mild hypertension	NR	Post-SARS-CoV-2 infection (on day 14 during her hospitalization for COVID-19 infection)	Since day 14: mild confusion with fluctuation of the mental status; day 16: myoclonic jerks and non-convulsive status epilepticus with coma	Suspected autoimmune encephalitis	Died
19	Duong 2020 and Huang 2020, USA [38,39]	1	41, F	RT-PCR positive	T2DM and obesity	NR	Initial presentation	Seizure, lethargy, disorientation, agitation, and hallucination	SARS-CoV-2-mediated encephalitis	Mental status improved by hospital day 12
20	Durovic 2021, Germany [40]	1	22, M	RT-PCR positive	Healthy	NR	Post-SARS-CoV-2 infection (10 days after SARS-CoV-2 infection)	Severe headache, neck stiffness, general weakness, and a loss of smell and taste	Autoimmune encephalitis	Discharged with full recovery
21	Efe 2020, Turkey [41]	1	35, F	RT-PCR positive	NR	NR	Initial presentation	Headache, nausea, dizziness, and drug-refractory seizures	SARS-CoV-2-mediated encephalitis	NR
22	El Aoud 2021, France [42]	1	60, M	Serology test positive	Dyslipidemia	Mild	Initial presentation	Headache, disturbance of consciousness, and vertigo	SARS-CoV-2-mediated encephalitis	Discharged and recovered after 1 month
23	Elmouhib 2022, Morocco [43]	1	54, F	RT-PCR positive	Healthy	Severe	Initial presentation	Altered mental state, dyspnea, altered consciousness	SARS-CoV-2-mediated autoimmune limbic encephalitis	Discharged with improved state
24	Esmaeili 2022, Iran [44]	1	67, M	RT-PCR positive	Healthy	Severe	Initial presentation	Drowsiness, decreased level of consciousness, deep tendon reflexes were brisk, and plantar reflexes were upward	Acute disseminated encephalitis	Died
25	Etemadifar 2020, Iran [45]	1	51, M	RT-PCR positive	Hypothyroidism and migraine	Mild	Initial presentation	For 3 days, episodic headache, nausea, and drowsiness, and generalized tonic-clonic seizure	SARS-CoV-2-mediated encephalitis	Died
26	Ferdosian 2021, Iran [46]	1	7, M	RT-PCR positive	Controlled seizures	Mild	Initial presentation	Loss of consciousness, inability to speak	SARS-CoV-2-mediated encephalitis	Discharged with supportive treatment
27	Freire-Álvarez 2020, Spain [47]	1	39, M	RT-PCR positive	NR	Mild	Initial presentation	Drowsiness, mental disorientation, inconsistent language disorder, and headache	SARS-CoV-2-mediated encephalitis	Following intravenous immunoglobulins and cytokine blockade with IL-6 receptor antagonist, the patient fully recovered after 30 days from admission
28	Gaughan 2021, Ireland [48]	1	16, F	RT-PCR positive	Mild learning disability	Asymptomatic	Initial presentation	Visual and auditory hallucinations, cognitive difficulties, and high-frequency tremor	Autoimmune encephalitis	Discharged; at six months, showed significant improvements
29	Ghosh 2020, India [49]	1	44, F	RT-PCR positive	Healthy	NR	Initial presentation	For 10 days: hypogeusia, hyposmia; then, confusion, disorientation, cognitive disorders, apraxia; then, tonic-clonic seizure and coma	SARS-CoV-2-mediated AHNE	Died
30	Grimaldi 2020, France [50]	1	72, M	RT-PCR positive	Transient global amnesia	Mild	Initial presentation	Since day 17: action tremor, ataxia, dysarthria, and upper limb dysmetria and myoclonus	Autoimmune encephalitis	Improved and discharged at day 37
31	Gunawardhana 2021, Sri Lanka [51]	1	47, F	RT-PCR positive	Uncomplicated T2DM	Mild	Post-SARS-CoV-2 infection (4 weeks after SARS-CoV-2 infection)	Confusion and abnormal behavior, seizures, status epilepticus	SARS-CoV-2-mediated encephalitis	Discharged to home with only minor residual cognitive deficits
32	Haider 2020, USA [52]	1	66, M	RT-PCR positive	Benign prostatic hypertrophy, fatty liver disease, and hypertension	NR	Initial presentation	Seizure; impaired orientation to time, place, and person; and persistent confusion	SARS-CoV-2-mediated encephalitis	Two months post-discharge, the patient showed significant improvements following rituximab
33	Hassan 2021, Pakistan [53]	1	58, M	RT-PCR positive	Hypertension	Severe	Initial presentation	Acute chorea	SARS-CoV-2-mediated encephalitis	Discharged
34	Hayashi 2020, Japan [54]	1	75, M	RT-PCR positive	Mild Alzheimer’s disease	Severe	Initial presentation	Left-dominant kinetic tremor in hands, alerted consciousness, dysmetria, ataxia, disorientation, and mild gait disturbance	SARS-CoV-2-mediated encephalitis	Died
35	Kahwagi 2021, Senegal [55]	1	7, F	RT-PCR positive	NR	Mild	Post-SARS-CoV-2 infection (On day 6 during her hospitalization)	Initial: headache; day 6: generalized tonic-clonic seizures; day 9: gait and behavioral disturbance, confusional syndrome, osteotendinous, and hyperreflexia	SARS-CoV-2-mediated encephalitis	Complete recovery over the follow-up of 2 months
36	Kamal 2020, United Arab Emirates [56]	1	31, M	RT-PCR positive	Healthy	Mild	Initial presentation	Day 3, behavioral disturbance; day 5: altered mental state, acute behavioral changes, severe confusion, fluctuations in the level of consciousness, and drowsiness	SARS-CoV-2-mediated encephalitis	Discharged; further follow-up confirmed good condition
37	Kasturiarachi 2022, USA [57]	1	19, F	RT-PCR positive	Menorrhagia, learning disability, and remote suicidal ideation	NR	Post-SARS-CoV-2 infection (recent infection)	Headaches, vomiting, and psychosis, left gaze deviation and right hemiplegia, unable to follow commands or open her eyes spontaneously, seizures, hyperreflexia in the right upper and lower extremities, no hyperkinetic movements	Sjogren’s-associated encephalitis	Discharged with improved mental status but needed to be monitored closely as an outpatient
38	Khoo 2020, UK [58]	1	65, F	RT-PCR positive	Alzheimer’s disease, osteoarthritis, and gastro-esophageal reflex disease	Mild	Post-SARS-CoV-2 infection (2 weeks after SARS-CoV-2 infection)	Week 2: widespread involuntary movements, diplopia, cognitive decline, speaking difficulties, increasing confusion; at entry: myoclonus, ocular movement disorder, aphasia, and perseveration	Autoimmune encephalitis	Discharged with improved neurological symptoms; back to baseline after 1 month from onset
39	Kimura 2021, Japan [59]	1	68, F	RT-PCR positive	Hypertension	NR	Post-SARS-CoV-2 infection (2 weeks after SARS-CoV-2 infection)	Her eyes were fixed in position and complete flaccid paralysis with diminished tendon reflexes in all extremities; no pathological reflex	Autoimmune encephalitis (SARS-CoV-2-mediated Bickerstaff encephalitis)	Discharged with residual double vision and bilateral disturbance in abduction
40	Koh 2022, Republic of Korea [60]	1	20, F	RT-PCR positive	Healthy	Moderate	Initial presentation	Tonic-clonic seizure On her left face and arm, drowsy mentality, personality change, dizziness, and somnolence	Suspected autoimmune encephalitis	Discharged with near-complete recovery
41	Kumar 2020, India [61]	1	35, M	RT-PCR positive	Headache	NR	Initial presentation	10 days before: headache; at entry: coma	SARS-CoV-2-mediated ANE	Died
42	Kumar 2022, India [62]	1	9 days, neonate	RT-PCR positive	Respiratory distress, hypoxia	NR	Post-SARS-CoV-2 infection (MRI done after 42 days of illness)	Generalized hypotonia generalized seizures	SARS-CoV-2-mediated encephalitis	Discharged with tachypnoea without hypoxia
43	Marques 2022, Portugal [63]	2	49, F	RT-PCR positive	Healthy	NR	Post-SARS-CoV-2 infection (6 days after SARS-CoV-2 infection)	Altered mental status, lethargic, not oriented to time and place, could not follow commands, neck rigidity, amnesia	SARS-CoV-2-mediated encephalitis	Two months post discharge, she was doing well, with no neurological signs and symptoms
			50, F	RT-PCR positive	Depression	NR	Post-SARS-CoV-2 infection (8 days after SARS-CoV-2 infection)	Restless, sometimes physically aggressive, mutism		Three months post discharge, she was doing well with no neurological signs and symptoms
44	McAbee 2020, USA [11]	1	11, M	RT-PCR positive	Healthy	Asymptomatic	Initial presentation	Status epilepticus	SARS-CoV-2-mediated encephalitis	Recovered within 6 days
45	Mekheal 2022, USA [64]	1	88, F	NR	Hypertension	NR	Post-SARS-CoV-2 infection (2 months after SARS-CoV-2 infection)	Right leg weakness, dysarthria, altered mental status	Autoimmune encephalitis	NR
46	Meshref 2021, Egypt [65]	1	66, F	RT-PCR positive	Chronic bronchitis and ischemic heart disease	NR	Initial presentation	Delirious state, confusion, fluctuant conscious level, and disorientation	SARS-CoV-2-mediated encephalitis	Discharged home with full consciousness, no neurological deficits
47	Mierzewska-Schmidt 2022, Poland [66]	1	2 months, Boy	RT-PCR positive	Healthy	NR	Initial presentation	Irritability, nystagmus, agitation then apathy	SARS-CoV-2-mediated AHNE	The baby showed signs of brain death
48	Miqdad 2021, Saudi Arabia [67]	1	36, M	RT-PCR positive	Glucose-6 phosphate dehydrogenase deficiency	NR	Initial presentation	Cognitive impairment and decreased responsiveness	SARS-CoV-2-mediated encephalitis	Discharged home with regular follow-up in the neurology clinic
49	Monti 2020, Italy [68]	1	50, M	RT-PCR positive	Mild hypertension	Asymptomatic	Initial presentation	Confabulations and delirious ideas; day 4: impaired awareness and status epilepticus	Autoimmune encephalitis	Discharged after 4 months of hospitalization without neurological deficits
50	Moriguchi 2020, Japan [17]	1	24, M	RT-PCR positive	NR	Mild	Initial presentation	Headache, consciousness disturbance, generalized seizures, and status epilepticus	SARS-CoV-2-mediated encephalitis	NR
51	Morvan 2020, France [69]	1	56, M	RT-PCR positive	Malnutrition, renal lithiasis with left renal abscess and *Mycobacterium abscessus* pulmonary infection	NR	Initial presentation	Coma	SARS-CoV-2-mediated ANE	Died
52	Mullaguri 2021, USA [70]	2	77, F	RT-PCR positive	Parkinson’s disease, cognitive impairment, and hypertension	Severe	Initial presentation	Oriented to self but not to place or time	SARS-CoV-2-mediated AHNE	Died
			68, F	RT-PCR positive	Chronic lymphocytic leukemia and hypertension	Severe	Post-SARS-CoV-2 infection (5 days after SARS-CoV-2 infection)	Comatose, persistent severe encephalopathy		Died
53	Natarajan 2020, India [10]	1	13, F	RT-PCR positive	Healthy	Mild	Initial presentation	Headache and generalized tonic-clonic seizure	SARS-CoV-2-mediated encephalitis	Discharged home in a normal neurological state
54	Oosthuizen 2021, South Africa [71]	1	52, M	RT-PCR positive	Healthy	NR	Initial presentation	Multidirectional gaze-evoked nystagmus, dysarthria, and truncal and appendicular ataxia	SARS-CoV-2 mediated encephalitis	Discharged while walking independently, mild emotional lability persisted
55	Orsini 2021, Brazil [72]	1	52, M	RT-PCR positive	Healthy	Severe	Initial presentation	Intense agitation, cognitive impairment, tonic-clonic seizure	SARS-CoV-2 mediated encephalitis	Died
56	Panariello 2020, Ecuador [73]	1	23, M	NR	Healthy	Moderate	Initial presentation	Psychomotor agitation, anxiety, thought disorganization, persecutory delusions, dyskinesias and auditory hallucinations	Autoimmune encephalitis	Clinical condition improved
57	Picod 2020, France [74]	1	58, F	Serology test positive	Hypertension and chronic kidney disease	Asymptomatic	Post-SARS-CoV-2 infection	Clonic seizure, aphasia, right-side hemiparesis, coma, and myoclonus	SARS-CoV-2-mediated encephalitis	Discharged from intensive care unit on day 17, with mild short-term memory impairment
58	Pilotto 2020, Italy [75]	1	60, M	RT-PCR positive	Healthy	Mild	Initial presentation	First 5 days: irritability, confusion, and asthenia; day 4–5: cognitive fluctuation, at entry: severe akinetic syndrome, mutism, and inhibited, archaic reflexes	SARS-CoV-2-mediated encephalitis	Discharged with normal neurological features
59	Pizzanelli 2021, Italy [76]	1	74, F	RT-PCR positive	Mild hypothyroidism	Severe	Initial presentation	Day 13: mild confusion and brief episode of impaired awareness; day 14: generalized tonic-clonic seizure	SARS-CoV-2-mediated autoimmune limbic encephalitis	Discharged on day 35
60	Poursadeghfard 2021, Iran [77]	1	18, F	RT-PCR positive	Healthy	NR	Initial presentation	Blurred vision, drowsy	SARS-CoV-2-mediated encephalitis	NR
61	Rebeiz 2020, USA [78]	1	30 s, M	RT-PCR positive	History of alcohol abuse	Asymptomatic	Initial presentation	1^st^ admission: confusion, behavioral changes, psychotic features including hallucinations; 2nd admission: worsened mental status, non-verbal, progressive neurological deterioration, and seizures	SARS-CoV-2-mediated encephalitis	Died
62	Reddy 2021, USA [79]	1	22, F	RT-PCR positive	Infantile seizures	Asymptomatic	Initial presentation	2 days of headache; at entry: acute altered mental status; while hospitalized: status epilepticus	SARS-CoV-2-mediated encephalitis	Died
63	Sangare 2020, France [80]	1	56, M	RT-PCR positive	Hypertension	Severe	Initial presentation	Vegetative state	SARS-CoV-2-mediated encephalitis	Discharged after 5.5 months, with mild attention deficit disorder
64	Sarmast 2022, Pakistan [81]	1	63, F	RT-PCR positive	Hypothyroidism and diabetes mellitus	NR	Initial presentation	Confusion accompanied by restlessness, fearfulness, and visual hallucinations. She was anxious, agitated, and aggressive. Altered level of consciousness, slight tremors of the limbs, and psychomotor restlessness	SARS-CoV-2-mediated encephalitis	Two weeks post discharge, she was doing well, with no neurological signs and symptoms
65	Sattar 2020, Pakistan [82]	1	44, M	RT-PCR positive	Healthy	Moderate	Initial presentation	Day 20: generalized tonic-clonic seizures and confusion	SARS-CoV-2-mediated encephalitis	Discharged on day 34 with normal neurological state
66	Sharma 2022, USA [83]	3	43, M	RT-PCR positive	Healthy	Mild	Initial presentation	Bitemporal headache, tonic-clonic seizures,	Self-limiting hemorrhagic encephalitis	Discharged with occasional headaches
			43, M	RT-PCR positive	Healthy	Severe	Post-SARS-CoV-2 infection (5 days after SARS-CoV-2 infection)	Non-verbal, and had an episode of rapid eye fluttering and gaze deviation, acute respiratory distress syndrome	Self-limiting leukoencephalopathy	Discharged but complained about recurrent headaches
			52, M	RT-PCR positive	Diabetes, hypertension, and hyperlipidemia	Severe	Post-SARS-CoV-2 infection (3 weeks after SARS-CoV-2 infection)	Bifrontal headache, blurred vision, left- and right-sided ptosis, ischemic third and sixth nerve palsy	SARS-CoV-2-mediated encephalitis	Discharged with assistance. No light perception in the left eye with complete ophthalmoplegia, intact vision in the right eye with ptosis
67	Sofijanova 2020, Republic of Macedonia [84]	1	9 months, infant	RT-PCR positive	NR	Severe	Initial presentation	Tonic-clonic seizures, disturbed consciousness, shortness of breath, weakened reaction to painful stimuli	NR	NR
68	Svedung Wettervik 2020, Sweden [85]	1	40′s, F	RT-PCR positive	Healthy	Severe	Initial presentation	Coma	SARS-CoV-2-mediated AHLE	After TPE treatment for 5 days, patient showed clinical and biochemical improvements
69	Tee 2022, Malaysia [86]	1	69, M	RT-PCR positive	Hypertension and atrial fibrillation	NR	Initial presentation	Altered behavior	SARS-CoV-2-mediated encephalitis	Subsequently remained well, with no neurological sequelae
70	Urso 2022, Italy [87]	1	5, F	RT-PCR positive	Healthy	NR	Initial presentation	Neck swelling, right latero-cervical and painful lymphadenopathy, altered mental status, and drowsiness	SARS-CoV-2-mediated encephalitis	Discharged when COVID-19 test came back negative
71	Valadez-Calderon 2022, Mexico [88]	1	28, M	NR	No history of chronic disease	Mild	Post-SARS-CoV-2 infection (2 weeks after SARS-CoV-2 infection)	Incoherent speech, somnolence, auditory hallucinations, suicidal ideation, and generalized tonic-clonic seizures	Autoimmune encephalitis (anti-NMDAR and anti-glutamic acid decarboxylase 65 co-expression)	Discharged home, but after six weeks-follow-up, he continues physical rehabilitation and presents neurological sequelae related to mood changes, irritability, and agitation episodes
72	Vandervorst 2020, Belgium [89]	1	29, M	RT-PCR positive	Healthy	Moderate	Initial presentation	Confusion, disorientation in time and space, immediate and short-term memory deficits, concentration and attention difficulties, anxiety, paranoid delusions, followed by dysgeusia and anosmia	Suspected SARS-CoV-2-associated encephalitis	Improved during hospitalization
73	Woldie 2020, Canada [90]	1	24, M	RT-PCR positive	AIHA	Severe	Post-SARS-CoV-2 infection (one week later at his follow-up appointment)	Persistent headache, decreased level of consciousness, and seizure activity.	SARS-CoV-2-mediated ANE	Died
74	Ye 2020, China [91]	1	NR, M	RT-PCR positive	NR	Moderate	Initial presentation	Confusion, altered consciousness	SARS-CoV-2 associated encephalitis	Discharged with cleared consciousness
75	Zambreanu 2020, UK [92]	1	66, F	RT-PCR positive	NR	Mild	Initial presentation	Confusion, seizure, disoriented to time and place, amnestic and mild word-finding difficulties	Limbic encephalitis	Neurological recovery
76	Zandifar 2020, Iran [93]	2	49, M	Not performed	NR	Severe	Initial presentation	Seizures; disorientation to place, time, and persons; and decrease of consciousness	Suspected SARS-CoV-2 associated encephalitis	Died
			39, M	RT-PCR positive		Moderate		Disoriented, agitated headache, tonic seizure, decreased consciousness and non-responsive verbal or painful commands	SARS-CoV-2 associated encephalitis	
77	Zanin 2021, Italy [12]	1	47, M	RT-PCR positive	Healthy	Mild	Initial presentation	Intense headache, epileptic seizures	SARS-CoV-2 associated encephalitis	Died
78	Zuhorn 2020, Germany [94]	1	54, M	RT-PCR positive	Arterial hypertension, obesity (BMI 34 kg/m^2^), obstructive sleep apnea syndrome	Moderate	Initial presentation	Aggressiveness, Disorientation, and stupor	Parainfectious encephalitis	Recovered and was discharged with only mild cognitive impairment

Abbreviations: NR: not reported, M: male, F: female, T2DM: type 2 diabetes mellitus, AHLE: acute hemorrhagic leukoencephalitis, AIHA: autoimmune hemolytic anemia (AIHA), TPA: tissue plasminogen activator, AHNE: acute hemorrhagic necrotizing encephalitis, ANE: acute necrotizing encephalitis.

**Table 2:** Diagnostic features of encephalitis in patients with COVID-19.

No.	Study ID	Neuroimaging		Neurophysiology	Serum Analysis	CSF Analysis					Other Pathogen Investigation
		Brain CT Scan Result	Brain MRI Result	EEG Result		WBC	Total Protein (mg/dL)	Glucose (mg/dL)	SARS-CoV-2	Other Explorations	
1	Ahsan 2021	NR	Axial T2 showed left perirolandic cortex and posterior parietal lobe cerebral edema, and axial DWI showed restricted diffusion.	Cerebral slowing with left focal slowing	MOG IgG positive	Elevated	48	46	NR	OCB positive	Bacterial or viral pathogens were negative
2	Allahyari 2021	Generalized brain edema	Generalized brain edema	NR	CRP 2+	Elevated	241	55	Positive	Anti-NMDAR Positive, HSV 1 and HSV 2 DNA negative	NR
3	Andrea 2020	Presented with non-specific diffuse cortical atrophy	NR	Triphasic waves were observed	Normal CRP and LDH, severe hyponatremia	Normal	61	49	Negative	None	HSV negative
4	Ayatollahi 2020	Normal	1st admittance: normal; 2nd admittance: signal hyperintensities on FLAIR and T2-weighted sequences in the claustrum bilaterally	1st admittance: slow wave activity; 2nd admittance: moderate bilateral non-epileptiform abnormalities	Thrombocytopenia, normal RBC, WBC and hemoglobin, CRP. ANA, aPL, aCL, anti-dsDNA, and ANCA were negative	Elevated	30	41	Negative	None	HSV negative
5	Ayuningtyas 2022	NR	No lesion or intracerebral or intracerebellar pathological enhancement was found	NR	Increased CRP level, and increased D-dimer level	Elevated	108	63	Positive	No Bacteria and AFB	Anti-HIV, HbSAg, anti-HCV, HSV, and CMV negative
6	Ayuso 2020	Normal	2nd admittance: hyperintense lesions in the caudal vermis and right flocculus, and contrast enhancement was observed in the floor of the fourth ventricle	Normal	Normal	Normal	41	70	NR	OCB, anti-Hu, anti-Yo, anti-Ri, anti-CV2, anti-Ma2, and anti-amphiphysin abs were negative	Anti-GD1 was positive. HIV, VZV, EBV, CMV, and *Mycoplasma* *Pneumoniae* were negative
7	Babar 2020	Normal	Normal	Generalized slowing	Elevated CRP, ferritin, TPO ab, and D-dimer. Negative anti-NMDAR ab, anti-GAD ab, VGKC ab, ANA, ANCA, IgM anti-β2-GPI ab, anti-DNase B ab, anti-streptolysin ab, IL-1 β, IL-6, IL-10, IL-2, C3, and C4	Normal	18	74	Negative	None	Gram bacteria negative
8	Benameur 2020	NR	Cerebral hemispheric restricted diffusion and cerebral edema	NR	Increased levels of anti-S1 IgM; anti-E IgM, IL-6, IL-8, IL-10, IP-10, and TNF-α	Elevated	>200	40	Negative	None	Influenza A virus positive, influenza B virus negative
		NR	Splenium lesion and FLAIR recovery	Diffuse slowing		Normal	37	111	Negative	None	Bacterial or viral pathogens were negative
		NR	Equivocal fluid-attenuated inversion recovery, FLAIR abnormality in the right temporal lobe	NR		Normal	21	88	Negative	None	Bacterial or viral pathogens were negative
9	Bernard-Valnet 2020	NR	Normal	Nonconvulsive, focal status epilepticus, slowed theta background rhythm	NR	Mild elevated	46.6	NR	Negative	None	Bacterial or viral pathogens were negative
		NR	Normal	NR	NR	Mild elevated	46.1	NR	Negative	None	
10	Bhavsar 2020	Normal	NR	Slow background without epileptiform discharges or seizures	Normal CBC, CRP and ESR, negative autoimmune encephalopathy antibody panel, hyponatremia	Elevated	173	35	Negative	None	Bacterial or viral pathogens were negative
11	Bodro 2020	Normal	Normal	NR	Elevated D-dimer	Elevated	105.5	80	Negative	Elevated IL-1β, IL-6, ACE	Bacterial or viral pathogens were negative
		Normal	Normal	NR	Elevated CRP, ferritin, LDH, and D-dimer, mild platelet reduction	Elevated	115.5	54	Negative	Elevated IL-6, ACE	NR
12	Burr 2021	NR	Normal	NR	Normal CRP and ESR and positive NMDAR-IgG positivity	NR	25	56	Negative	None	Bacterial or viral pathogens were negative
13	Cao 2020	NR	Bilateral hyperintense lesions in the deep and periventricular supratentorial white matter, either punctiform and slightly diffuse (cases 1–3) or diffuse and confluent (cases 4 and 5), associated with lesions in the pons for two patients (cases 1 and 2)	Unspecific slow-wave activity	Elevated IL-6	normal	32	NR	Negative	Normal IL-6	NR
					Normal IL-6		26		Negative	Normal IL-6	
					Elevated IL-6		115		Negative	Elevated IL-6	
					Elevated IL-6		18		Negative	None	
					Elevated IL-6		18		Negative	Normal IL-6	
14	Casez 2021	NR	Hyperintensity of the olfactory tracts on T2 fluid-attenuated inversion recovery, and diffusion-weighted imaging	NR	NR	8 WBC	Normal	NR	Negative	None	NR
15	Chalil 2020	Extensive bilateral parietal and occipital intraparenchymal hemorrhage and extensive edema causing hydrocephalus	Cortical gadolinium enhancement with hyper-intense T2 and FLAIR signal surrounding the hemorrhages	Mild diffuse slowing	Elevated D-dimer, CRP, and ferritin	Elevated	NR	NR	Negative	Elevated CSF IgG ratio	Negative for VZV, HSV, and ENV
16	Cheraghali 2021	NR	Symmetric, cortical, and juxtacortical high T1 and T2 signal abnormality, in bilateral parieto-occipital lobes	NR	Elevated level of blood sugar, AST, ALT, ESR, LDH, and positive CRP test	Normal	15	100	Positive	Negative for bacterial growth	HSV 1 and HSV 2 negative
17	Dahshan 2022	Normal	Normal	NR	NR	Normal	Normal	Normal	NR	HSV 1 and HSV 2 negative	NR
18	Dono 2021	NR	Axial T2 fluid-attenuated inversion recovery (T2-FLAIR) and axial diffusion-weighted imaging showed hyperintense lesions of the bilateral parietal cortex, left temporal cortex, and right cingulate cortex	Epileptiform abnormalities, continuous sharp waves and spike-and-slow-wave complexes	Slight lymphocytopenia, elevated D-dimer, normal CRP	Elevated	47	78	Negative	OCB positive	HSV, EBV, CMV, and VZV were negative
19	Duong 2020 and Huang 2020	Normal	NR	Generalized slowing with no epileptic discharges	Normal	Elevated	100	120	Positive	None	Negative for bacterial culture and HSV 1
20	Durovic 2021	NR	Multiple disseminated pathological T2 and FLAIR hyperintensities	NR	Lyme borreliosis and HIV was negative, MOG antibody positive	Elevated	39.9	64	Negative	HSV 1 and HSV 2 negative	Complete recovery over the follow-up of 2 months
21	Efe 2020	NR	Hyperintense signal in the left temporal lobe in T2 and T2 FLAIR	NR	NR	NR	NR	NR	NR	None	NR
22	El Aoud 2021	Normal	Focal hyperintense signal in the splenium of the corpus callosum on T2 FLAIR and diffusion-weighted images	Slow oscillations without epileptiform features	Lymphophenia, elevated CRP and ferritin, hypoalbuminemia, ANA, and ANCA were negative	normal	49	55	NR	None	*Mycoplasma pneumoniae*, syphilis, HIV, influenza A and B were negative
23	Elmouhib 2022	Normal	High-signal intensity lesion on DWI, T2 FLAIR in the temporal lobes, without diffusion restriction on apparent diffusion coefficient map	NR	CRP at 200 mg/L with a negative PCT at 0.05 μg/L, ferritin at 2300 μg/L	Normal	100	63	NR	NR	NR
24	Esmaeili 2022	NR	Extensive high signal lesions in T2W and FLAIR sequences on bilateral cerebral hemispheres, para-ventricular and subcortical white matter, middle cerebellar peduncles, centrum semi vale, corpus callosum, basal ganglia, thalami, midbrain, and pons. Post-contrast MRI showed sparse enhancements on midpart of the midbrain and left parietal lobe	NR	Elevated CRP and ESR, prothrombin time and partial thromboplastin time were normal	Normal	Normal	Normal	Negative	EBV, HSV, CMV, VZV negative	NR
25	Etemadifar 2020	Generalized brain edema and signs of brain herniation	Generalized brain edema, downward herniation of cerebellar tonsils and brain stem, and FLAIR hyperintensities in bilateral cerebral cortices and corpus striatum	Normal	Leukocytosis, lymphopenia, elevated D-dimer	NR	NR	NR	NR	None	NR
26	Ferdosian 2021	NR	Diffuse brain edema	NR	CPK: 42, LDH: 554, CRP: weakly +, ESR: 6. COVID-19 PCR was negative	Normal	30	57	Positive	HSV, Enterovirus negative	NR
27	Freire-Álvarez 2020	Normal	Extensive involvement of the brain, including cortical and subcortical right frontal regions, right thalamus, bilateral temporal lobes and cerebral peduncles, with no leptomeningeal enhancement	NR	Elevated ferritin, IL-6, and D-dimer	Elevated	198	48	Negative	None	CMV, HSV 1 and 2, human HHV 6, HPeV, and VZV negative
28	Gaughan 2021	NR	Two tiny punctate T2/FLAIR hyper-intensities in the centrum semiovale bilaterally	Delta slowing	Autoimmune antibody panel negative	NR	43	52.2	Negative	Autoimmune antibody panel negative	HSV and VZV negative
29	Ghosh 2020	NR	Non-enhancing altered intensity lesions in the left high fronto-parietal and right posterior parietal areas with peri-lesional edema; isolated cortical venous thrombosis	NR	All blood parameters normal	Elevated	60	70	Negative	Elevated IgG index, and OCB negative	Bacterial or viral pathogens were negative
30	Grimaldi 2020	NR	Normal	Symmetric diffuse background slowing	Elevated fibrinogen and CRP, IgG autoantibodies extremely high	Normal	49	NR	Negative	OCB negative and IgG autoantibodies	NR
31	Gunawardhana 2021	Bi-frontal white matter oedema	T2 FLAIR hyperintensities in the periventricular white matter, mainly clustered around frontal and occipital horns. FLAIR hyperintensities were also noted in the splenium, basal ganglia, and ventral pons	Low wave discharges consistent with encephalitis	Hemoglobin, liver function tests, creatinine and electrolytes were within normal limits, inflammatory markers (ESR, CRP) were mildly elevated	Elevated	Normal	Normal	SARS-CoV-2 IgM and IgG antibodies are positive SARS-CoV-2 RNA negative	HSV 1 and HSV 2, Japanese encephalitis, VZV were negative	NR
32	Haider 2020	Normal	Small acute/subacute lacunar infarcts and a patchy area of T2 bright signals in the cortical and periventricular regions, consistent with cerebritis	Global cerebral dysfunction and severe toxic metabolic encephalopathy	Autoimmune antibody panel negative	Normal	77	86	Negative	None	Bacterial or viral pathogens were negative
33	Hassan 2021	NR	Mild periventricular ischemic changes	NR	Increased CRP, D-dimer, and serum ferritin	Normal	NR	NR	Positive	HSV, OCBs were negative	NR
34	Hayashi 2020	NR	Abnormal hyperintensity in the splenium of corpus callosum on diffusion-weighted image	NR	Elevated CRP, lymphopenia	NR	NR	NR	NR	None	NR
35	Kahwagi 2021	NR	Normal	Overall slowing of the pattern with the presence of diffuse pseudoperiodic complexes predominating in fronto-temporal area	Elevated CRP	Normal	76	NR	NR	None	NR
36	Kamal 2020	Multiple hypodensities in the external capsules bilaterally, the insular cortex, and the deep periventricular white matter of the frontal lobes bilaterally	Abnormal signal intensity in the temporal lobe cortex bilaterally in a rather symmetrical fashion. In addition, the involvement of the parasagittal frontal lobes bilaterally was evident as well, displaying bright signals on T2-fluid-attenuated inversion recovery and T2-weighted images with corresponding diffusion restriction	Did not display any significant epileptic discharges, possibly due to the masking effect of lorazepam	Elevated D-dimer	Normal	55	67	Positive	Normal LA, RF, ANA and aCL	*Mycobacterium* *Tuberculosis*, Gram bacteria, HSV, HHV, and VZV were negative
37	Kasturiarachi 2022	NR	Contrast-enhancing lesion in the left occipital, temporal, and frontal lobes, the vermis folia, and tectal plate colliculi; hyperperfusion on arterial spin labeling in the left hemisphere	Left hemispheric poly spike and waves	Elevated LDH, reticulocyte count and bilirubin, schistocytes, and low haptoglobin. Positive ANA, high anti-SSA (anti-Ro) and anti-SSB (anti-La) antibodies, and significantly elevated COVID-19 antibody	NR	NR	NR	Negative	Meningitis/encephalitis panel negative	NR
38	Khoo 2020	NR	Normal	Normal	Elevated CRP and D-dimer	Normal	Normal	Normal	Negative	Anti-NMDAR ab and A panel of antineuronal abs and OCB were negative	NR
39	Kimura 2021	NR	No significant abnormalities	No evidence of seizure activity nor response to photic and sound stimuli	Seropositive for anti–SARS-CoV-2 antibodies	Normal	20	164	Negative	OCBs positive	*Campylobacter* *jejuni*, *Haemophilus influenzae*, *Mycoplasma pneumoniae,* cytomegalovirus, and EBV negative
40	Koh 2022	Patchy ground-glass opacities on bilateral lung fields, compatible with COVID-19 pneumonia	Diffuse cortical high signal intensities, especially on bilateral insula with increased arterial spin labeling signals	Repeated high-amplitude polymorphic delta activities from the right frontotemporal area evolving to generalized 1–2 Hz spike-wave discharges, suggesting an impending focal status epilepticus	IL-6 was mildly elevated to 21.7 pg/mL, CRP level normal	Elevated	NR	NR	Negative	Elevated IL-6	HSV, VZV, enterovirus, tuberculosis, EBV, toxoplasmosis, and syphilis negative
41	Kumar 2020	Hypodensities in both thalami and left caudate nucleus; left parasellar-middle cranial fossa mass	Left parasellar-middle cranial fossa mass (MRI was performed about 2 weeks earlier than CT)	NR	Leukocytosis	Normal	Elevated	NR	NR	None	HSV and VZV were negative
42	Kumar 2022	NR	Subcortical volume loss (right occipital and left parieto occipital) with cystic changes, tiny hemorrhages at the caudothalamic groove with loss of myelination at the posterior limb of internal capsule	Normal	COVID Ig G And Ig M antibodies were positive	Normal	Normal	Normal	NR	NR	NR
43	Marques 2022	Normal	Did not show any pathological changes	Moderate encephalopathy	Elevated LDH and d-dimers	Elevated	82	59	Negative	HSV, VZV, cytomegalovirus negative	HSV, HIV, VZV negative
		Normal	Did not show any pathological changes	Mild encephalopathy, without epileptiform activity	HSV 1–2, HIV, and VZV Negative	Normal	16	93	Negative	HSV, VZV, cytomegalovirus negative	NR
44	McAbee 2020	Negative	NR	Frontal intermittent delta activity	NR	Mild elevated	97	92	NR	None	NR
45	Mekheal 2022	Without contrast, an old left cerebellar infarct, with no evidence of acute infarct or hemorrhage	Old infarct, acute infarct involving the left cerebellum, as well as an effacement of the left temporal horn and edema within the left pons, midbrain, left temporal lobe, and surrounding the basal ganglia	Moderate-severe diffuse encephalopathy without epileptiform discharges or seizures	Elevated ESR, CRP, and D-dimer, serum COVID-19 IgG antibody positive	Elevated	145	75	Negative	Meningitis/encephalitis panel by PCR were negative, including all microbial cultures	NR
46	Meshref 2021	Right temporal hypo-dense area	Ill-defined area of faint low signal intensity lesion in T1, hyperintense in T2. FLAIR images showed partial restriction in DWI with no significant enhancement post IV gadolinium contrast injection, involving the right cerebral hemisphere, mainly at the temporal area, suggesting encephalitis	NR	NR	NR	Normal	Normal	NR	No bacterial growth, herpes virus was negative	NR
47	Mierzewska-Schmidt 2022	NR	Diffuse areas of oedema associated with numerous symmetrical changes with punctate hemorrhages in basal ganglia, thalami, brainstem, and cerebral gray matter	NR	Low Hb 9.3 g/dL and Platelet count 183 × 10^3^/μL, CRP 7.4 mg/L, D-dimers 0.97	Elevated	660.00	< 10	Positive	Elevated lactic acid, meningoencephalitis PCR panel was negative	All bacterial cultures were negative
48	Miqdad 2021	Unremarkable	Normal	Different abnormalities suggestive of encephalitis	CRP, D-dimer, and procalcitonin were high	Elevated	832	2.59	NR	HSV PCR and gram stain negative	NR
49	Monti 2020	NR	Normal	Abnormal	Elevated levels of IL-6	Elevated	NR	NR	NR	OCB and anti-NMDAR ab positive, with elevated levels of IL-6 and IL-8	Bacterial or viral pathogens were negative
50	Moriguchi 2020	Normal	Diffusion -weighted images showed hyperintensity along the wall of inferior horn of right lateral ventricle. FLAIR images showed hyperintense signal changes in the right mesial temporal lobe and hippocampus with slight hippocampal atrophy	NR	Elevated levels of WBC and CRP	Mild elevated	NR	NR	Positive	NR	HSV and VZV were negative
51	Morvan 2020	Acute hydrocephalus with diffuse cerebral edema, spontaneous bilateral thalamic hyperdensities, with discrete contrast enhancement and spontaneous hyperdensity in subarachnoidal spaces.	Hypersignal of both thalami brainstem and cerebellum with some hemorrhagic component on T2 sequences	NR	Elevated levels of CRP, fibrinogen, and D-dimer Low Hb, high AST, low factor V, high troponin, high creatinine, very low kaliemia	Normal	79	NR	Negative	None	HIV negative
52	Mullaguri 2021	Axial sections of the brain showed punctate hemorrhages in the right frontal and left frontal and parietal areas	Axial section showed hyperintensities in bilateral centrum semiovale areas. MRI of the brain showing innumerable punctate microhemorrhages in the cerebellar peduncles and subcortical regions of bilateral hemispheres, including bilateral basal ganglia and internal capsules	NR	Hyponatremia (132 mMol/L), significant elevations in D-dimer, LDH, ferritin, CRP, and CK	NR	NR	NR	NR	NR	NR
53	Natarajan 2020	NR	Normal	Normal	NR	Elevated	86	77	Negative	None	HSV, CMV, and VZV were negative
54	Oosthuizen 2021	Central midbrain hypodensity	Features consistent with brainstem encephalitis	Normal	Elevated ESR	Elevated	37	65	Positive	Immunoglobulin G index 0.62, SARS-CoV-2 antibody negative	Tests for infections and malignancy negative
55	Orsini 2021	NR	Normal	Normal	NR	Normal	60	53	Positive	Bacterial culture negative	NR
56	Panariello 2020	Normal	NR	Theta activity at 6 Hz	Elevated CRP and D-dimer with negative ANA, ANCA, anti-ENA, aCL, and anti-β2-GPI abs	NR	65.4	70	Negative	Elevated IL-6 and anti-NMDAR ab	HSV, EBV, CMV, VZV, and enterovirus were negative
57	Picod 2020	Normal	Bilateral lesions (hypersignal or enhancement of meninges, cortical and subcortical regions spread over the insula, the cingula, the medial part of occipital areas, and the internal part of the left-side temporal lobe)	Diffuse intermittent periodic activity	Moderately elevated IL-6	Normal	28	NR	Negative	Elevated IL-6 and OCB negative	HSV negative
58	Pilotto 2020	Normal	Normal	Generalized slowing, with decreased reactivity to acoustic stimuli	Elevated D-dimer, a wide immunological screening of immune-mediated encephalitis was negative	Mild elevated	69.6	NR	Negative	Slightly elevated IL-6, strongly elevated IL-8, TNF-α and β2M	Neurotropic viruses negative
59	Pizzanelli 2021	Normal	Bilateral symmetrical mesial temporal lobes T2/FLAIR/DWI hyperintensities, with mild hippocampal thickening	Autoimmune panel for encephalitis negative	Elevated CRP and fibrinogen	Normal	104	67	Negative	OCB and autoimmune panel for encephalitis negative	Neurotropic viruses negative
60	Poursadeghfard 2021	NR	FLAIR increased signal intensity in the cortical and subcortical regions of both mesial temporal lobe as well as both side hippocampal tails, with relative symmetrical appearance without evidence of significant enhancement or restricted DWI compatible with viral or autoimmune encephalitis	NR	NR	NR	NR	NR	NR	NR	Cat-scratch disease, toxoplasmosis, syphilis, Lyme disease, brucellosis, HIV, VZV, HSV, CMV, EBV, and hepatitis B and C were negative
61	Rebeiz 2020	A questionable subarachnoid hemorrhage within the mesial parietal region and nonspecific hypoattenuation in the splenium of the corpus callosum	1st MRI: DWI and FLAIR hyperintensity of the splenium of corpus callosum; 2nd MRI (after readmission): new abnormal T2/FLAIR hyperintense and restricted diffusion involving the left thalamus, right parasagittal frontal cortex, and bilateral genu of the corpus callosum	Generalized slowing	VES 27; Normal IL-6 and CRP	Elevated	297	56	Negative	None	Neurotropic viruses negative
62	Reddy 2021	4 days after admission: severe diffuse cerebral edema with cerebellar tonsillar herniation	No acute intracranial process	NR	NR	Normal	108	88	NR	None	Bacterial or viral pathogens were negative
63	Sangare 2020	NR	Multiple small hemorrhagic lesions in the pontine tegmentum, bilateral subinsular region	Poorly reactive delta slow waves	NR	Normal	Normal	Normal	NR	None	NR
64	Sarmast 2022	Unremarkable	Hyperintense signals in frontoparietal and parietotemporal lobes on FLAIR/T2 sequence	NR	Mildly elevated CRP, elevated LDH, CPK, ferritin, and D-dimer	Normal	66	81	Negative	VZV PCR, HSV 1–2 PCR, CMV PCR, bacterial antigen negative	Negative for HSV 1–2, HIV, enterovirus, and VZV virus
65	Sattar 2020	Day 20: few scattered foci of white matter hypo-attenuation	Day 25: abnormal medial cortical signals in the bilateral frontal lobe region	NR	Autoimmune antibody panel negative	Mild elevated	39	75	Positive	CSF color pinkish	Bacterial or viral pathogens were negative
66	Sharma 2022	left temporal hypodensity	Intense focal edema within the left hippocampus with mild restricted diffusion, postcontrast enhancement, and hemorrhage seen on susceptibility-weighted imaging	Normal	Creatinine of 1.3 mg/dL, CRP of 42.4 mg/L, ESR of 95 mm/h, CK 858 IU/L, D-Dimer of 1821 FEU, fibrinogen of 644 mg/dL, ferritin of 1352.9 ng/mL, LD of 392 IU/L, prothrombin time (PT/INR) of 1.3/15	Elevated	44	59	NR	Gram stain, bacterial culture, and meningitis/ encephalitis panel were negative. VZV PCR, cryptococcal antigen, culture, AFB smear and culture, and CMV PCR were negative. VZV IgG elevated	NR
		Small right temporal hyperdensity (0.6 cm diameter) suggestive of a hemorrhage with normal vasculature	Right anterior temporal lobe intraparenchymal hemorrhage; additional multiple scattered foci of susceptibility artifact particularly in the gray–white junctions and corpus callosum; and sulcal FLAIR hyperintensity in the right frontal, biparietal, and left temporal lobes	NR	Elevated IL-6, fibrinogen, and thrombocytopenia	Elevated	118	28	NR	Meningitis/encephalitis panel, AFB smear and culture, cryptococcal antigen, and VZV PCR, CMV PCR, CSF cytology were negative	NR
		1st admission: stenosis of the cavernous segment of the right ICA. 2nd admission: unremarkable	1st admission: mild periventricular white matter disease. 2nd admission: multiple punctate foci of restricted diffusion in the bilateral frontal, parietal, occipital, and temporal lobes, mild meningeal enhancement of the anterior and middle cranial fossa, and opacification of multiple ethmoid and mastoid air cells	NR	1st admission; creatinine of 1.4 mg/dL, ESR 111 mm/h, CRP 43.5 mg/L, and HbA1c 12.6%. 2nd admission: fibrinogen of >1000 mg/dL, ferritin of 743 ng/mL, ESR of >130 mm/h, CRP of 313 mg/L, PT/INR of 19/1.6, procalcitonin of 1.24 ng/mL, and antithrombin-3 activity of 62%	Elevated	82	78	NR	Borrelia burgdorferi IgM/IgG titer (0.09 LIV, 0.09 LIV), negative CSF ACE, VDRL, and cryptococcal antigen, EBV PCR positive	AIDP, myasthenia gravis, or vasculitis, secondary infections, mycoplasma IgM, anti-cardiolipin antibodies, B-2 glycoprotein, anti-Xa essay, HIV antibodies, hepatitis panel, serum cryoglobulin PAVAL, autoimmune encephalitis panel were negative
67	Sofijanova 2020	Enlargement of the lateral ventricles, intraventricular masses, pronounced internal hydrocephalus.	NR	NR	Normal	NR	202.7	91.4	NR	None	NR
68	Svedung Wettervik 2020	White matter brain edema with compressed convexity sulci	Increased white matter intensity on flair images, microhemorrhages with involvement of basal ganglia on susceptibility-weighted imaging	Pronounced, generalized slowing over both hemispheres, no electrographic epileptic activity	Mild increased IL-6	Elevated	NR	86.4	Negative	Elevated levels of IL-6	Negative for bacteria and neurotropic viruses
69	Tee 2022	Noncontrast brain CT revealed an old right lenticular infarct	Performed 1 month later, normal findings	Performed 3 weeks later, normal findings	NR	Elevated	116	NR	Positive	NR	NR
70	Urso 2022	NR	CNS involvement, suggestive of encephalitis	A slow base rhythm (theta-delta) together with synchronous bilateral potentials formed by slow waves with predominance on the right side.	NR	Normal	27	Normal	Negative	No bacterial or tuberculous infection	HSV1, varicella-zoster, EBV, and CMV were negative
71	Valadez- Calderon 2022, Mexico	NR	Hyperintensities in the bilateral anterior cingulate cortex and temporal lobes	Subcortical dysfunction in frontal, temporal, and occipital regions	Normal	Normal	Normal	Normal	Negative	NR	NR
72	Vandervorst 2020	Normal	Asymmetric FLAIR hyperintensity of the left medial temporal cortex associated with mild gyral expansion	General excess of beta-rhythm	NR	Normal	Normal	Normal	Negative	None	Negative for enterovirus and HSV
73	Woldie 2020	Non-specific symmetric cerebral enhancement	T2/FLAIR hyper intensity on the medial aspect of each temporal lobe, bilateral basal ganglia, and medial thalami consistent with severe acute necrotizing encephalitis	NR	Elevated LDH	NR	NR	NR	NR	NR	Positive for *Cryptococcus neoformans*
74	Ye 2020	Normal	NR	NR	Low WBC and lymphocytes	Normal	27	56.5	negative	None	Negative for bacterial or tuberculous infection
75	Zambreanu 2020	Normal	Non-enhancing, symmetrical T2 and FLAIR hyperintensities in mesial temporal lobes and medial thalami and to a lesser extent upper pons, as well as scattered subcortical white matter hyperintensities	NR	Elevated CRP, lymphopenia	Normal	100	63	Negative	Autoimmune antibody panel negative	Negative for streptococci, meningococcus, haemophilus, listeria, *Escherichia coli*, HSV 1 and 2, HHV6, enteroviruses, parechovirus, CMV, VZV, and Cryptococcus
76	Zandifar 2020	Diffuse brain parenchymal edema and reduced lateral ventricles	NR	NR	Leukocytosis and lymphopenia	Mild elevated	70	30	Negative	None	NR
		NR			Leukocytosis and lymphopenia and LDH elevated	Mild elevated	74	33	Positive		
77	Zanin 2021	Increasing vasogenic oedema in the right temporo-fronto-parietal region with extension to the capsular region, to the cerebral peduncle and in the ipsilateral mesencephalic region. Severe compressive effect on the right lateral ventricle	Severe vasogenic oedema of the white matter with 10 mm shift of the midline and compression of the right lateral ventricle; DWI: extensive cortical marked restriction	NR	Increased CRP and WBC with lymphopenia	NR	NR	NR	NR	None	NR
78	Zuhorn 2020	NR	Signal alterations within the claustrum/external capsule region, showed reduced diffusion	NR	LDH, D-dimer, myoglobin, IL-6 and CRP, IgA and IgG positive for SARS-CoV-2	Mild elevated	39.6	57	Negative	None	Negative for HSV, VZV, cytomegaly and Epstein Barr virus

Abbreviation: NR: not reported, AFB: acid-fast bacilli, CT: computed tomography, WBC: white blood cells, RBC: red blood cell, MOG: myelin oligodendrocyte glycoprotein, AST: aspartate aminotransferase, ALT: alanine aminotransferase, HSV: herpes simplex virus, CRP: C-reactive protein, LDH: lactate dehydrogenase, FLAIR: fluid-attenuated inversion recovery, ANA: antinuclear antibody, aCL: anticardiolipin antibody, dsDNA: double-stranded DNA, ANCA: antineutrophil cytoplasmic antibodies, NMDAR: N-methyl-D-aspartate receptor, GAD: glutamic acid decarboxylase, VGKC: voltage gated potassium channel, IL: interleukin, GPI: glycoprotein I, ab: antibody, TPO: thyroid peroxidase, IP: induced protein, CBC: complete blood count, ESR: erythrocyte sedimentation rate, CPK: creatine phosphokinase, CK: creatine kinase, VZV: varicella-zoster virus, ENV: erythrocytic necrosis virus, EBV: Epstein–Barr virus, CMV: cytomegalovirus, OCB: oligoclonal band, DWI: diffusion-weighted imaging, HIV: human immunodeficiency virus, HHV: human herpes virus, HPeV: human parechovirus, LA: lupus anticoagulant, RF: rheumatoid factor, ENA: extractable nuclear antigens, β2M: β2-microglubulin. Normal protein level: 15–45 mg/dL, normal glucose level: 40–70 mg/dL.

## Data Availability

The data presented in this study are available within the article and Supplementary Materials.

## Supplementary Materials

The following supporting information can be downloaded at: https://www.mdpi.com/article/10.3390/cells11162575/s1, Table S1: Search strategies, Table S2: Laboratory features of encephalitis patients with SARS-Cov-2 infection, Table S3: Quality assessment of the included case reports, Table S4: Quality assessment of the included case series.
