# Supplementary material for: Encephalitis in Patients with COVID-19: A Systematic Evidence-Based Analysis
Source: Cells. 2022 Aug 18;11(16):2575. doi: 10.3390/cells11162575 (PMC9406394; doi:10.3390/cells11162575)
Supplement: Supplementary file 1 [file cells-11-02575-s001.zip › cells-1830327-supplementary.pdf]

Table S1. Search strategies

Databases	Search strategies
**PubMed**	(Coronavirus[Title] OR COVID-19[Title] OR COVID19[Title] OR nCoV[Title] OR SARS-CoV-2[Title] OR SARS-CoV2[Title]) AND (encephalitis[Title])<br>Year restriction 2019-2022
**Scopus**	TITLE(Coronavirus OR COVID-19 OR COVID19 OR nCoV OR SARS-CoV-2 OR SARS-CoV2) AND TITLE(encephalitis)<br>Year restriction 2019-2022
**Google Scholar**	allintitle: ("Coronavirus" OR "COVID-19" OR "COVID19" OR "nCoV" OR "SARS-CoV-2" OR "SARS-CoV2") ("encephalitis")<br>Year restriction 2019-2022

Table S2. Laboratory features of encephalitis patients with SARS-Cov-2 infection

No.	Study ID	Leukocyte types	Opening pressure when performing lumbar puncture (Ref: 100–200 mm H_2O)	Serum blood glucose value	IgG index
1	Ahsan 2021	**Hospital Admission 1**<br>WBC 132 cells/mm^3<br>Lymphocytes 64%<br>**Hospital Admission 2 (+7d)**<br>WBC 116 cells/mm^3<br>Lymphocytes 90%	NR	NR	0.55
2	Allahyari 2021	WBC 13900, Neutrophils: 87%,<br>Lymphocytes: 10%	NR	NR	NR
3	Andrea 2020	NR	NR	NR	NR
4	Ayatollahi 2020	Increased WBC (20 cells/uL, 100% lymphocytes)	150 mm	Normal	NR
5	Ayuningtyas 2022	Leukocytes 17900/UL, Neutrophils: 84%<br>Lymphocytes: 10.1%	NR	104 mg/dl	NR
6	Ayuso 2020	0/mm^3	NR	NR	0.5
7	Babar 2020	NR	NR	NR	NR
8	Benameur 2020	Neutrophils: 51% (0%–6%) Lymphocytes: 10% (40%–80%)<br>Macrophages: 39% (15%–45%)	300 mm	NR	NR
		Neutrophils: 75% (0%–6%)	480 mm		

		Lymphocytes: 25% (40%–80%)<br>Macrophages: 0% (15%–45%)			
		Neutrophils: 0% (0%–6%)<br>Lymphocytes: 0% (40%–80%)<br>Macrophages: 0% (15%–45%)	120 mm		
9	Bernard-Valnet 2020	Lymphocytes: 97%	NR	NR	NR
		Lymphocytes: 89%			
10	Bhavsar 2020	Increased WBC (122 cells/uL) with mononuclear cells predominance	NR	109 mg/dL	NR
11	Bodro 2020	Lymphocytic pleocytosis (95 cells/uL, 98% lymphocytes, 2% macrophages)	NR	NR	NR
		Lymphocytic pleocytosis			
12	Burr 2021	WBC 7 cells/mL (89% lymphocytes and 11% monocytes)	NR	105 mg/dL	NR
13	Cao 2020	NR	NR	NR	NR
14	Casez 2021	WBC 8 cells/mm^3 (75% polymorphonuclear neutrophilic cells)	NR	NR	NR
15	Chalil 2020	Nucleated cells: 76 x 10^6/L (65% neutrophils)	NR	NR	IgG ratio 0.35 (Ref: 0-0.23) IgG index 1.05 (Ref: 0.25-0.85)
16	Cheraghali 2021	WBC 14700 cells/µl, Lymphocytes: 20%	NR	NR	NR
17	Dahshan 2022	NR	NR	NR	NR
18	Dono 2021	Lymphocytosis (26 cells/mm^3)	NR	NR	NR
19 & 20	Duong 2020 and Huang 2020	100% lymphocytes	NR	200 mg/dL	NR

21	Durovic 2021	NR	NR	NR	NR
22	Efe 2020	NR	NR	NR	NR
23	El Aoud 2021	WBC 1 cell/mm^3	NR	NR	NR
24	Elmouhib 2022	Lymphocytes 360 cells/uL	NR	112 mg/dl	NR
25	Esmaeili 2022	Lymphopenia	NR	NR	NR
26	Etemadifar 2020	NR	NR	NR	NR
27	Ferdosian 2021	WBC 14000/mm^3, Neutrophils:71%, Lymphocytes: 21%	NR	NR	NR
28	Freire-Álvarez 2020	Leukocyte 20 cells/mL (90% lymphocytes)	NR	NR	NR
29	Gaughan 2021	Normal WBCs and lymphocyte count	NR	NR	NR
30	Ghosh 2020	Mild lymphocytosis (total 20 cells, including 90% lymphocytes)	NR	NR	Elevated
31	Grimaldi 2020	NR	NR	NR	NR
32	Gunawardhana 2021	NR	NR	Normal	NR
33	Haider 2020	WBC 3 cells/mm^3	150 mm	NR	NR
34	Hassan 2021	Increased Leukocyte count, High Neutrophils	NR	NR	NR
35	Hayashi 2020	NR	NR	NR	NR
36	Kahwagi 2021	NR	NR	NR<br>182 mg/dL	NR
37	Kamal 2020	WBC <5 cells/mm^3	NR	88 mg/dL	NR
38	Kasturiarachi 2022	Lymphocytes: 51%	NR	104 mg/dl	NR

		Neutrophils: 46.1%<br>Monocytes: 1%<br>Eosinophils: 1.9%			
39	Khoo 2020	WBC 1 cell/mm^3	NR	NR	NR
40	Kimura 2021	NR	NR	NR	NR
41	Koh 2022	NR	NR	NR	NR
42	Kumar 2020	NR	NR	NR	NR
43	Kumar 2022	NR	NR	NR	NR
44	Marques 2022	NR	170 mm	NR	NR
45	McAbee 2020	16 WBCs, Neutrophils: 8% (0%-6%)	NR	NR	NR
46	Mekheal 2022	WBC: 10.8x10^3 cells/mm^3	NR	NR	NR
47	Mierzewska-Schmidt 2022	Lymphocytes: 42%<br>Neutrophils: 52%	NR	149 mg/dl	NR
48	Miqdad 2021	WBC: 302 cells/uL, Lymphocytes: 85%,<br>Neutrophils: 15%	NR	NR	NR
49	Monti 2020	WBC 76 (type NR)	NR	NR	IgG link index<br>1.45 (Ref: <0.70)
50	Moriguchi 2020	WBC 12 cells/mL	>320 mm	NR	NR
51	Morvan 2020	0 leucocytes	NR	NR	NR
52	Mullaguri 2021	Lymphocytes: 23300 cells/mm^3 (83%)	NR	NR	NR
53	Natarajan 2020	WBC 200 cells/mm^3, all lymphocytes	NR	126 mg/dL	NR
54	Oosthuizen 2021	NR	NR	NR	NR

55	Orsini 2021	Lymphocytes: 50%<br>Monocytes: 12%<br>Neutrophils: 4%	NR	NR	NR
56	Panariello 2020	NR	NR	NR	NR
57	Picod 2020	NR	NR	NR	NR
58	Pilotto 2020	Mild lymphocytic pleocytosis (18/µl)	NR	NR	NR
59	Pizzanelli 2021	Normal WBC count	NR	NR	NR
60	Poursadeghfard 2021	NR	NR	NR	NR
61	Rebeiz 2020	Lymphocytic pleocytosis with WBC 350 cells/µl, 98% mononuclear cells.	NR	NR	NR
62	Reddy 2021	WBC 1 cell/uL	NR	NR	NR
63	Sangare 2020	NR	NR	NR	NR
64	Sarmast 2022	NR	NR	NR	NR
65	Sattar 2020	Total nucleated cells count 11; eosinophil 1%, segmented neutrophils 75%, lymphocytes 21%, monocytes/macrophages 3% with mild pleocytosis	NR	NR	NR
66	Sharma 2022	Case 1: WBC: 15470 cells/mm^3<br>Case 2: WBC: 12000 cells/mm^3<br>Case 3: WBC: 9940 cells/mm^3 (Neutrophils: 80%)	NR	NR	NR
67	Sofijanova 2020	NR	NR	242.3 mg/dL	NR
68	Svedung Wettervik 2020	Increase in leukocytes (21 x 10^6/L polynuclear and 25 x 10^6/L mononuclear)	NR	NR	NR
69	Tee 2022	NR	95 mm	NR	NR
70	Vandervorst 2020	NR	NR	NR	NR
71	Woldie 2020	NR	NR	NR	NR

72	Urso 2022	NR	NR	NR	NR
73	Valadez-Calderon 2022	NR	NR	NR	NR
74	Ye 2020	WBC 1 cell/mm^3	220 mm	NR	NR
75	Zambreanu 2020	WBC 3 cells/mm^3	NR	NR	0.48
76	Zandifar 2020	11 WBC	NR	NR	NR
		9 WBC			
77	Zanin 2021	NR	NR	NR	NR
78	Zlotnik 2022	NR	NR	NR	NR
79	Zuhorn 2020	9 WBC (88% Lymphocytes)	NR	NR	NR

Table S3. Quality assessment of the included case reports

No.	Study ID	Quality assessment checklist for case reports								Yes (%)
		1	2	3	4	5	6	7	8	
1	Ahsan 2021	Y	Y	Y	Y	Y	Y	Y	Y	100.0
2	Allahyari 2021	Y	Y	Y	Y	Y	Y	Y	Y	100.0
3	Andrea 2020	Y	Y	Y	Y	Y	Y	Y	Y	100.0
4	Ayatollahi 2020	Y	Y	Y	Y	Y	Y	Y	Y	100.0
5	Ayuningtyas 2022	Y	Y	Y	Y	Y	Y	Y	Y	100.0
6	Ayuso 2020	Y	Y	Y	Y	Y	Y	Y	Y	100.0
7	Babar 2020	Y	Y	Y	Y	Y	Y	Y	Y	100.0
8	Bernard-Valnet 2020	Y	Y	U	Y	Y	Y	Y	Y	87.5
9	Bhavsar 2020	Y	Y	Y	Y	Y	Y	Y	Y	100.0
10	Bodro 2020	Y	Y	Y	Y	Y	U	N	Y	75.0
11	Burr 2021	Y	Y	Y	Y	Y	Y	N	Y	87.5
12	Casez 2021	Y	Y	N	Y	N	N	N	N	37.5
13	Chalil 2020	Y	Y	Y	Y	Y	Y	Y	Y	100.0
14	Cheraghali 2021	Y	Y	Y	Y	Y	Y	Y	Y	100.0
15	Dahshan 2021	Y	Y	Y	Y	Y	Y	Y	U	87.5
16	Dono 2021	Y	Y	Y	Y	Y	Y	N	U	75.0
17	Duong 2020	Y	Y	Y	Y	Y	Y	N	N	75.0
18	Durovic 2021	Y	Y	Y	Y	Y	Y	N	Y	87.5
19	Efe 2020	Y	Y	U	Y	Y	Y	N	Y	75.0
20	El Aoud 2021	Y	Y	Y	Y	Y	Y	Y	Y	100.0
21	Elmouhib 2022	Y	Y	Y	Y	Y	Y	N	Y	87.5
22	Esmaeili 2022	Y	U	Y	Y	Y	U	N	Y	62.5
23	Etemadifar 2020	Y	Y	Y	Y	Y	Y	N	Y	87.5
24	Ferdosian 2021	Y	Y	Y	Y	Y	Y	N	N	75.0
25	Freire-Álvarez 2020	Y	Y	Y	Y	Y	Y	Y	Y	100.0
26	Gaughan 2021	Y	Y	Y	Y	Y	Y	Y	N	87.5
27	Ghosh 2020	Y	Y	Y	Y	Y	Y	N	Y	87.5
28	Grimaldi 2020	Y	Y	Y	Y	Y	Y	Y	Y	100.0
29	Gunawardhana 2021	Y	Y	Y	Y	Y	N	N	Y	75.0
30	Haider 2020	Y	Y	Y	Y	Y	Y	Y	Y	100.0

31	Hassan 2021	Y	N	Y	Y	Y	Y	N	N	62.5
32	Hayashi 2020	Y	Y	Y	Y	Y	U	U	Y	75.0
33	Huang 2020	Y	Y	Y	Y	Y	Y	N	Y	87.5
34	Kahwagi 2021	Y	Y	Y	Y	Y	Y	U	N	75.0
35	Kamal 2020	Y	Y	Y	Y	Y	Y	Y	Y	100.0
36	Kasturiarachi 2021	Y	Y	Y	Y	Y	Y	N	Y	87.5
37	Khoo 2020	Y	Y	Y	Y	Y	Y	N	Y	87.5
38	Kimura 2021	Y	Y	Y	Y	Y	Y	N	Y	87.5
39	Koh 2022	Y	Y	Y	Y	Y	Y	N	Y	87.5
40	Kumar 2020	Y	Y	Y	Y	Y	Y	U	Y	87.5
41	Kumar 2022	Y	Y	Y	Y	Y	Y	U	Y	87.5
42	Marques 2022	Y	Y	Y	Y	Y	Y	Y	Y	100.0
43	McAbee 2020	Y	Y	Y	Y	NA	NA	NA	Y	100.0
44	Mekheal 2022	Y	U	Y	Y	Y	U	N	Y	62.5
45	Meshref 2021	Y	Y	Y	Y	Y	Y	N	Y	87.5
46	Mierzewska-Schmidt 2022	Y	Y	Y	Y	NA	NA	NA	U	80.0
47	Miqdad 2021	Y	Y	Y	Y	Y	Y	N	Y	87.5
48	Monti 2020	Y	Y	Y	Y	Y	Y	Y	Y	100.0
49	Moriguchi 2020	Y	N	Y	Y	Y	N	N	Y	62.5
50	Morvan 2020	Y	Y	Y	Y	Y	Y	N	Y	87.5
51	Mullaguri 2021	Y	Y	Y	Y	Y	Y	U	Y	87.5
52	Natarajan 2020	Y	Y	Y	Y	Y	Y	N	Y	87.5
53	Oosthuizen 2021	Y	Y	Y	Y	Y	U	N	U	62.5
54	Orsini 2021	Y	Y	Y	Y	U	U	NA	Y	71.4
55	Panariello 2020	Y	Y	Y	Y	Y	Y	N	Y	87.5
56	Picod 2020	Y	Y	Y	Y	Y	Y	Y	Y	100.0
57	Pilotto 2020	Y	Y	Y	Y	Y	Y	N	Y	87.5
58	Pizzanelli 2021	Y	Y	Y	Y	Y	U	N	Y	75.0
59	Poursadeghfard 2021	Y	Y	Y	Y	Y	Y	N	Y	87.5
60	Rebeiz 2020	Y	Y	Y	Y	Y	Y	N	Y	87.5
61	Reddy 2021	Y	Y	Y	Y	N	Y	N	Y	75.0
62	Sangare 2020	Y	Y	Y	Y	U	Y	N	Y	75.0
63	Sarmast 2022	Y	Y	Y	Y	Y	Y	N	Y	87.5

64	Sattar 2020	Y	Y	Y	Y	Y	Y	N	Y	87.5
65	Sofijanova 2020	Y	N	Y	Y	N	Y	N	Y	62.5
66	Svedung Wettervik 2020	Y	Y	Y	Y	Y	N	N	Y	87.5
67	Tee 2022	Y	Y	Y	Y	Y	Y	N	N	75.0
68	Urso 2022	Y	Y	Y	Y	NA	NA	NA	N	80.0
69	Valadez-Calderon 2022	Y	Y	Y	Y	Y	U	N	Y	75.0
70	Vandervorst 2020	Y	Y	Y	Y	Y	Y	N	Y	87.5
71	Woldie 2020	Y	Y	Y	Y	Y	U	N	Y	75.0
72	Ye 2020	Y	N	Y	Y	Y	Y	N	Y	75.0
73	Zambreanu 2020	Y	N	Y	Y	Y	Y	N	Y	75.0
74	Zanin 2021	Y	Y	Y	U	Y	Y	Y	Y	87.5
75	Zuhorn 2020	Y	Y	Y	U	Y	Y	N	Y	75.0

1. Were patient's demographic characteristics clearly described? 2. Was the patient's history clearly described and presented as a timeline? 3. Was the current clinical condition of the patient on presentation clearly described? 4. Were diagnostic tests or assessment methods and the results clearly described? 5. Was the intervention(s) or treatment procedure(s) clearly described? 6. Was the post-intervention clinical condition clearly described? 7. Were adverse events (harms) or unanticipated events identified and described? 8. Does the case report provide takeaway lessons?

Table S4. Quality assessment of the included case series

No.	Study ID	Quality assessment checklist for case series										Yes (%)
		1	2	3	4	5	6	7	8	9	10	
1	Benameur 2020	Y	N	Y	Y	Y	Y	Y	Y	N	U	70.0
2	Cao 2020	Y	Y	Y	N	Y	Y	Y	Y	N	N	70.0
3	Sharma 2022	Y	Y	Y	Y	Y	Y	Y	Y	U	Y	90.0
4	Zandifar 2020	Y	N	Y	N	Y	Y	Y	Y	N	N	60.0

1. Were there clear criteria for inclusion in the case series? 2. Was the condition measured in a standard, reliable way for all participants included in the case series? 3. Were valid methods used for identification of the condition for all participants included in the case series? 4. Did the case series have consecutive inclusion of participants? 5. Did the case series have complete inclusion of participants? 6. Was there clear reporting of the demographics of the participants in the study? 7. Was there clear reporting of clinical information of the participants? 8. Were the outcomes or follow up results of cases clearly reported? 9. Was there clear reporting of the presenting site(s)/clinic(s) demographic information? 10. Was statistical analysis appropriate?
